# High-Valent Intermediate Observed in a Cu-Based OER Electrocatalyst by *Operando* X‑ray Absorption Spectroscopy

**DOI:** 10.1021/acs.jpclett.5c00944

**Published:** 2025-06-14

**Authors:** Raul Garcia-Diez, Romualdus Enggar Wibowo, Elmar Kataev, Wilson Quevedo Garzon, Marianne van der Merwe, Daniel Duarte-Ruiz, Caterina Cocchi, Marcus Bär

**Affiliations:** † Interface Design, 28340 Helmholtz-Zentrum Berlin für Materialien und Energie GmbH (HZB), Berlin 12489, Germany; ‡ Institute of Physics, Carl von Ossietzky Universität Oldenburg, Oldenburg 26129, Germany; ¶ Energy Materials In-Situ Laboratory Berlin (EMIL), HZB, Berlin 12489, Germany; § Helmholtz Institute Erlangen-Nürnberg for Renewable Energy (HI ERN), Erlangen 91058, Germany; ∥ Dept. Chemistry and Pharmacy, Friedrich-Alexander-Universität Erlangen-Nürnberg, Erlangen 91058, Germany

## Abstract

Understanding the chemical transformations governing the oxygen evolution reaction (OER) in Cu-based electrocatalysts is critical for advancing cost-efficient alkaline water-splitting technologies. In this study, we employ synchrotron-based *operando* Cu L_3_-edge X-ray absorption spectroscopy (XAS) and potentiodynamic techniques to probe the key intermediate species involved in alkaline OER. Our findings reveal that this metastable species exhibits an electronic structure resembling high-valent Cu complexes, particularly those associated with the CuO_2_^–^ ion. Potentiodynamic measurements indicate that the high-valent intermediate emerges at potentials as low as 1.62 V_RHE_, coinciding with the oxidative process traditionally attributed to the Cu^2+^ ↔ Cu^3+^ redox transition, suggesting that the formation of the high-valent intermediate is directly linked to this redox process. This work provides valuable insights into the interplay between redox chemistry and catalytic performance in Cu-based OER electrocatalysts and provides further insights into the nature of the chemical species governing the oxygen evolution reaction mechanism.

## Graphical abstract

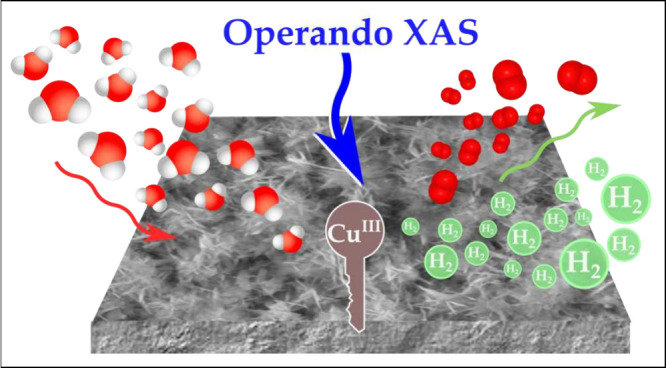

As the global push for decarbonization intensifies, the production of green hydrogen through water electrolysis has emerged as a pivotal technology for achieving clean and sustainable energy systems. Due to the sluggish kinetics of the oxygen evolution reaction (OER), the search for efficient and cost-effective OER electrocatalysts has generated significant research interest. Although precious metal-based materials, such as Ir and Ru oxides, remain the state-of-the-art OER catalysts,^1^ the need for cost reduction and scalability has driven the exploration of earth-abundant alternatives such as 3d transition metals. Among these, copper-containing catalysts have emerged as promising candidates in alkaline media,^2,3^ including CuO-based materials with OER activities^4,5^ comparable to more renowned approaches containing nickel or cobalt,^6,7^ which are, however, considered to be critical elements by the US Geological Survey.^8^ Hence, a deeper understanding of the reaction mechanism in copper-based electrocatalysts is essential for the knowledge-driven design of materials with enhanced OER activity and for developing strategies to mitigate their severe instability in high alkaline conditions, such as transpassive dissolution^9^ or corrosion.^10,11^

Intermediate species play a critical role in the OER pathways, and their identification during operation is crucial for understanding the active phase of copper-based electrocatalysts in alkaline media and the reasons underlying the catalyst’s degradation. High-valent Cu complexes are frequently identified as key intermediates in oxidative electrochemical reactions like glucose electrooxidation in nonenzymatic glucose sensors^12,13^ or OER. Cu^III^_2_O_3_, originally described by Müller,^14^ and Cu^III^OOH,^15^ in analogy with Ni^III^OOH, have been proposed as OER intermediates, with the former being recently suggested to decompose into Cu^II^O through oxygen release.^2^ Raman spectroscopic studies by Deng et al.^16^ under relevant operation conditions identified a Cu^III^O_2_^–^ species with a similar fingerprint to NaCu^III^O_2_, whereas Ostervold et al.^17^ highlighted hydroxide adsorption on the Cu^II^O surface (CuO-(OH*)_1/2_) as the critical intermediate. In contrast, Toparli et al.^9^ identified Cu_4_O_3_ as the most likely intermediate for the OER and subsequent transpassive dissolution. Complementarily, several calculated Pourbaix diagrams^18–20^ suggest the formation of a solvated hydroxide species, Cu^II^(OH)_4_^2–^, under these conditions. Conclusive elucidation of the reaction pathways of alkaline OER in copper-based electrocatalysts is still missing, and despite a broad consensus about the participation of a high-valent Cu complex, no element-specific direct method was used to probe the electronic structure of the intermediate under operating conditions and link it to the calculated structure.

To resolve these ambiguities, *operando* X-ray absorption spectroscopy (XAS) emerges as an ideal tool^21^ for probing intermediates that may otherwise evade detection due to their short lifetimes. The accumulation of an intermediate within the time scale accessible by *operando* methods requires that the OER intermediate stabilizes kinetically on the surface of the catalyst and becomes dominant, typically associated with the species at the rate-determining step in the mechanism pathway.^22^ In particular, XAS at the Cu L_2,3_-edge provides exceptional chemical sensitivity and insights into electronic transitions involving the active 3d-states.^23^ Synchrotron-based *operando* Cu L_2,3_-edge XAS is uniquely suited to capture the electronic configuration of Cu-based species under realistic electrochemical conditions with reasonable time resolution^24^ and can provide a deeper understanding of the hybridization of Cu 3d- and O 2p-states,^25^ which likely dictates the catalytic activity.

In this study, we employed *operando* Cu L_3_-edge XAS on a high surface area copper-based electrocatalyst under applied potentials relevant to alkaline OER to probe the elusive high-valent Cu intermediates. Prepared by the electrochemical anodizing process^26^ described in Materials and Experimental Methods in the , the investigated electrocatalyst presents a high surface-to-bulk ratio, as shown in the Scanning Electron Micrograph in Figure 1a. A high surface area is essential to maximize the spectroscopic signal from the electrochemically active species, considering that the measured signal is a convolution of the surface’s contribution and the inactive bulk-originated one, due to the large attenuation length of X-rays at the Cu L_3_-edge (around 80 nm for metallic copper).

**Figure 1:**
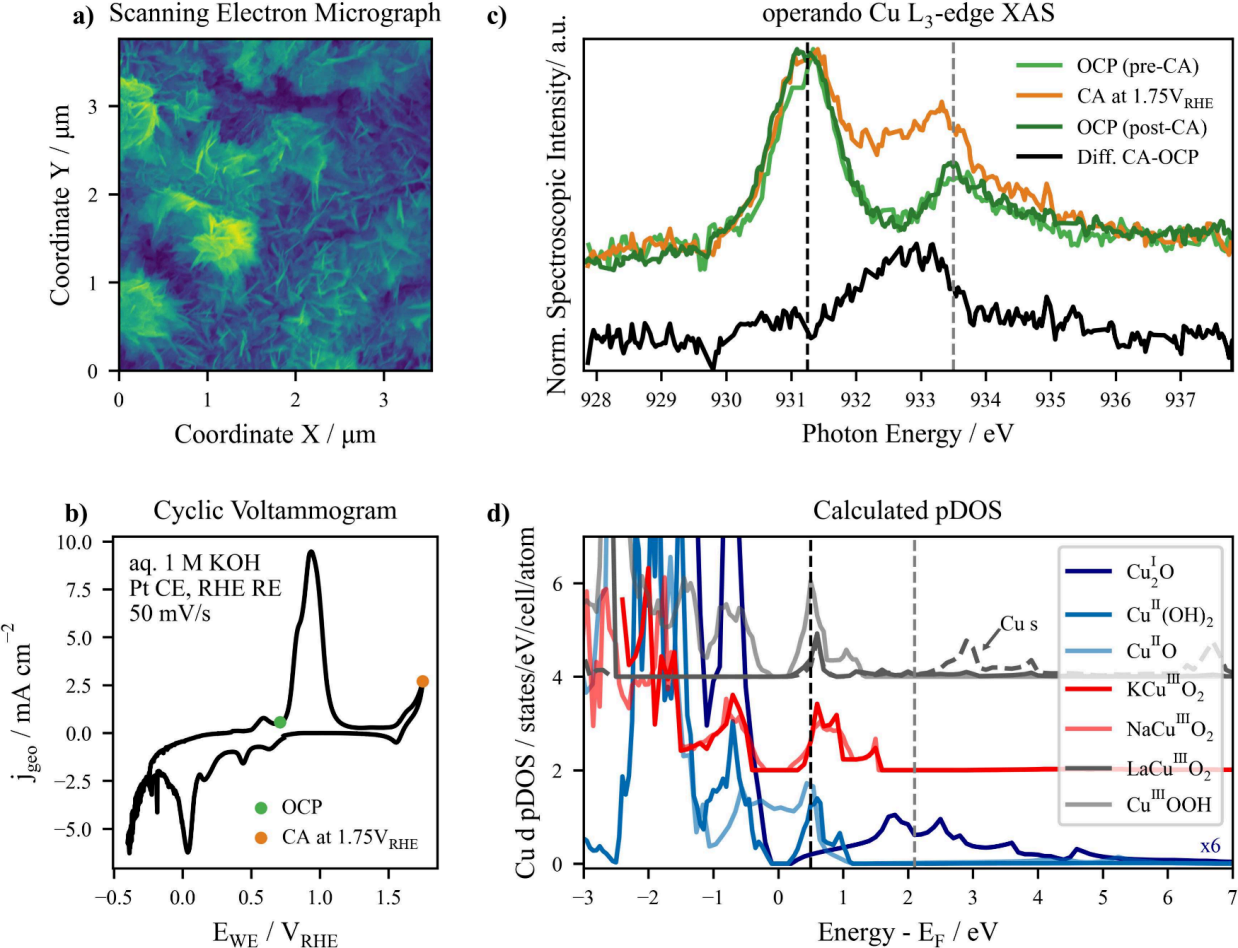
Characterization of the high surface area Cu-based electrocatalyst and speciation of the OER intermediate: (a) Scanning Electron Micrograph (SEM) of the catalyst. (b) Cyclic voltammogram (CV) of the catalyst in aq. 1 M KOH with a scan rate of 50 mV/s. (c) Normalized *operando* Cu L_3_-edge XAS of the catalyst under OER conditions (chronoamperometry (CA) at 1.75 V_RHE_) and at open circuit potential (OCP) before and after CA. The difference between the spectra measured at OCP (pre-CA, dark blue line) and during the CA at OER (red line) is shown below (black line). The (reversible) appearance of a peak around 932.6 eV only under applied potential above the OER onset hints at the formation of the sought-after OER intermediate. The vertical dashed lines indicate the peak energy of Cu^I^ and Cu^II^ oxides. (d) Calculated Cu d-states: Projected density of states (pDOS) calculated from DFT of complexes related to the electrocatalyst species at OCP (in blue, Cu^II^(OH)_2_, Cu^II^O, and Cu^I^_2_O multiplied by 6), of complexes potentially related to the OER intermediate species (KCu^III^O_2_ and NaCu^III^O_2_ in red), and of other high-valent Cu complexes (LaCu^III^O_2_ and Cu^III^OOH in gray). The Cu s-states of LaCu^III^O_2_ are shown for completeness. The vertical dashed lines indicate the LUMO energy of Cu^I^ and Cu^II^ oxides.

The studied electrocatalyst presents the characteristic cyclic voltammogram (CV) of copper in an alkaline medium, as depicted in Figure 1b for a measurement in aqueous 1 M KOH using the *operando* flow-through cell described by Garcia-Diez et al.^27^ CVs show a slight dependence on the sample’s history and the solution’s molarity, but the overall shape remains consistent. Below 1.3 V_RHE_, the 1 M KOH CV shows the redox peaks typically ascribed in its anodic branch to subsequent superficial oxidations of Cu to Cu^I^_2_O and to Cu^II^(OH)_2_ or Cu^II^O.^28,29^ Interestingly, a redox couple around 1.62 V_RHE_ in the forward cycling direction and 1.55 V_RHE_ in the reverse scan appears just below the potentials relevant for water oxidation operation. Although extensively attributed to the Cu^2+^ ↔ Cu^3+^ reaction,^30–32^ accurate identification of this redox process is essential for understanding the chemical transformations occurring during the OER regime. The potential of this Cu redox formation coincides with the onset of the OER-relevant potential regime; therefore, these *operando* investigations were made focusing on potentials in this region and above.

To understand the chemical transformation of the Cu-based electrocatalyst due to oxygen evolution, synchrotron-based Cu L_3_-edge absorption spectra are measured in the OÆSE endstation^27^ at the EMIL beamline in BESSY-II at Open Circuit Potential (OCP) conditions and at OER-relevant potentials, above 1.62 V_RHE_. Figure 1c shows the normalized data collected during chronoamperometry (CA) at 1.75 V_RHE_, as well as at the OCP before and after the CA measurement.

The spectrum measured at OCP before the CA experiment reveals two Cu species, Cu^I^_2_O and Cu^II^(OH)_2_/Cu^II^O, at 931.0 and 933.5 eV, respectively,^33–36^ arising from the anodization synthesis of the electrocatalyst, as previously reported^26,37^ and further confirmed by X-ray photoelectron spectroscopy (See Ex-situ characterization of the Cu-based electrocatalyst in the ). Upon applied potential (1.75 V_RHE_), the spectrum shows a new feature around 932.6 eV which lies between the Cu^I^ and Cu^II^ spectral features. Notably, this new feature disappears when returning to a relaxed state (OCP after CA. See Considerations about XAS at the OCP in the for more details about the relaxation behavior of the electrocatalyst). In fact, the spectrum recorded at the OCP after OER coincides with the data collected at the pristine OCP, prior to the CA at 1.75 V_RHE_. The reversibility of the spectral feature emerging only under OER-relevant potentials suggests the formation of a metastable species or intermediate.

In order to speciate the complex appearing under the OER conditions and compare its fingerprint with those of the Cu-based species previously proposed as the key OER intermediate, it is worth inspecting the difference between the spectra at the OCP (pre-CA) and during the CA at the OER conditions as shown at the bottom of Figure 1c, providing more insights into the shape of the transient spectral feature. The spectral fingerprint of the new species, centered at 932.6 ± 0.2 eV, does not lie close to the L_3_-edge energies associated with Cu^II^, Cu^I^ (dashed lines in Figure 1c), or Cu^0^ complexes (edge position similar to Cu^I^),^27^ suggesting that the new species possesses a different chemical environment. Comparison with reported organometallic and ternary complexes with formal Cu^III^ nature^38–41^ reveals that the peak energy of the probed intermediate falls within the range of values observed for these complexes (930–935 eV, associated with the Lowest Unoccupied Molecular Orbital (LUMO)), suggesting that its electronic structure is more similar to these high-valent Cu species.

The reversible mechanism governing the appearance of this OER intermediate resembles the reaction previously reported in some Cu^III^-hydroxide complexes.^42^ In this context, “reversibility” refers to the consistent buildup of each chemical species before and after the OER conditions, as seen in Figure 1c, rather than the restoration of the catalyst to its original state, as copper dissolution occurs inevitably at high applied potentials. Severe dissolution of the electrocatalyst is observed under these conditions, as highlighted by the complete loss of Cu-associated spectroscopic signal at the electrode after the experiment depicted in the microspectrographs shown in of the Supporting Information.

Although the previous comparison with Cu L_3_-edge energies reported in the literature points toward the formation of a high-valent Cu intermediate during OER, the wide range of Cu^III^ L_3_-edge peak energies arising from the strong Cu 3d-ligand hybridization^38^ (highlighting the ligand-driven nature of the LUMO) indicates the need for more detailed insights into the electronic structure of some Cu complexes. We can gain further understanding of the nature of these spectral features through *ab initio* calculations of projected density of states (pDOS) of Cu-based compounds with relevant oxidation states. Employing Density Functional Theory (DFT), the pDOSs of seven copper complexes were obtained as described in Materials and Experimental Methods in the , with their Cu d-states depicted in Figure 1d. As a good measure of the occupied and unoccupied states around the Fermi level, the Cu d-derived pDOS provides the basis for the spectral features probed experimentally. Specifically, the pDOS calculations include Cu^I^ and Cu^II^ complexes associated with the electrocatalyst’s surface species in its relaxed state, like Cu^I^_2_O, Cu^II^(OH)_2_, and Cu^II^O, as well as high-valent Cu complexes potentially related to the OER intermediate species, such as the ternary molecules KCu^III^O_2_, LaCu^III^O_2_, and NaCu^III^O_2_ or Cu^III^OOH. By inspecting the unoccupied Cu d-states above the Fermi Energy (E_F_), the presence of significant pDOS in Cu^II^(OH)_2_ and Cu^II^O around 0.5 eV can be associated with the spectral feature emerging around 931 eV in the *operando* Cu L_3_-edge XAS in Figure 1c, attributed to the presence of Cu^II^ species at the surface of the electrocatalyst at OCP. Analogously, the pDOS contribution between 1.7 and 2.8 eV in Cu^I^_2_O relates to the Cu^I^ species in the pristine electrocatalyst.

Interestingly, the calculations of the high-valent Cu complexes reveal the emergence of unoccupied Cu d-states at energies between both species, around 0.5 eV < E – E_F_ < 1.5 eV (Figure 1d), which can be related to the spectral feature associated with the OER intermediate probed under applied potential, at 932.6 eV. Specifically, the KCu^III^O_2_ and NaCu^III^O_2_ complexes exhibit the most significant pDOS contributions at this energy range, with a strongly localized state at 1.5 eV. Even considering the Cu s-derived states (shown exemplarily for LaCu^III^O_2_), only these two complexes depict abundant unoccupied electronic states in the energy region between the Cu^0^/Cu^I^ and Cu^II^ fingerprints. This observation supports the association of the spectral feature attributed to the OER intermediate with high-valent copper species, similar to KCu^III^O_2_ and NaCu^III^O_2_. Likely, the new probed species presents an electronic structure similar to the Cu^III^O_2_^–^ ion, present in both ternary complexes, as previously predicted.^16^

This combined experimental and theoretical analysis suggests that a high-valent copper species, likely similar to the Cu^III^O_2_^–^ ion, participates in the oxygen evolution reaction mechanism with a 1.75 V_RHE_ applied potential. However, it is still unclear if this intermediate species appears exclusively at OER-relevant potentials (above the OER onset, defined as ∼1.7 V_RHE_ in this study) or if it emerges already at lower potentials, specifically in the redox region around 1.62 V_RHE_. This question was addressed using a combination of Fixed-Energy X-ray Absorption Voltammetry (FEXRAV^43^) and Cyclic Voltammetry with varying upper potential limits (UPLs). With FEXRAV, the XAS signal at the photon energy associated with a distinct chemical species (the spectral fingerprint of the OER intermediate at 932, in this case) can be selectively monitored during potential-dependent measurements like Cyclic Voltammetry. Since the abundance of the chemical species in the electrocatalyst is proportional to the intensity of the collected spectroscopic signal, FEXRAV can shed light on the behavior of the monitored species and the dynamic processes dominating the electrochemical reaction.

The FEXRAV signal measured at 932.6 eV is shown in the top panel of Figure 2 while the bottom panel depicts the corresponding CVs. Collected for two different UPLs, the FEXRAV data reveal that the intermediate-related spectral fingerprint only appears for positive enough potentials, specifically, when the UPL is above the redox transition, coinciding with the potential regime where the OER occurs (see of the Supporting Information for more UPLs and scanning rates). When looking in more detail at the FEXRAV signal, it is evident that the spectroscopic intensity related to the new species, i.e., the abundance of the chemical species, grows for applied potentials above 1.62 V_RHE_ in the forward cycling direction (redox feature in the anodic branch, highlighted with a gray dashed line in Figure 2) and continues growing for higher potentials in the OER region until reaching a maximum at 1.55 V_RHE_ already in the backward direction (redox feature in the cathodic branch, gray dashed line), where the trend is reversed. At lower potentials in the reverse potential sweep, the abundance of this new species begins to decrease until its original concentration is reached around 1.0 V_RHE_. The increase in the FEXRAV signal, which only occurs at the OER-related potential window between the anodic and cathodic features of this redox transition, suggests that the oxidation process and chemical transformation occurring at this redox potential are directly linked to the formation of the OER intermediate. Importantly, there is no evidence that the oxidative process at the redox feature produces a distinct product or intermediate compared with what is observed under the OER conditions. Instead, the results align with prior findings in this study, indicating that the OER intermediate likely corresponds to an oxidized species with a high-valent Cu nature.

**Figure 2:**
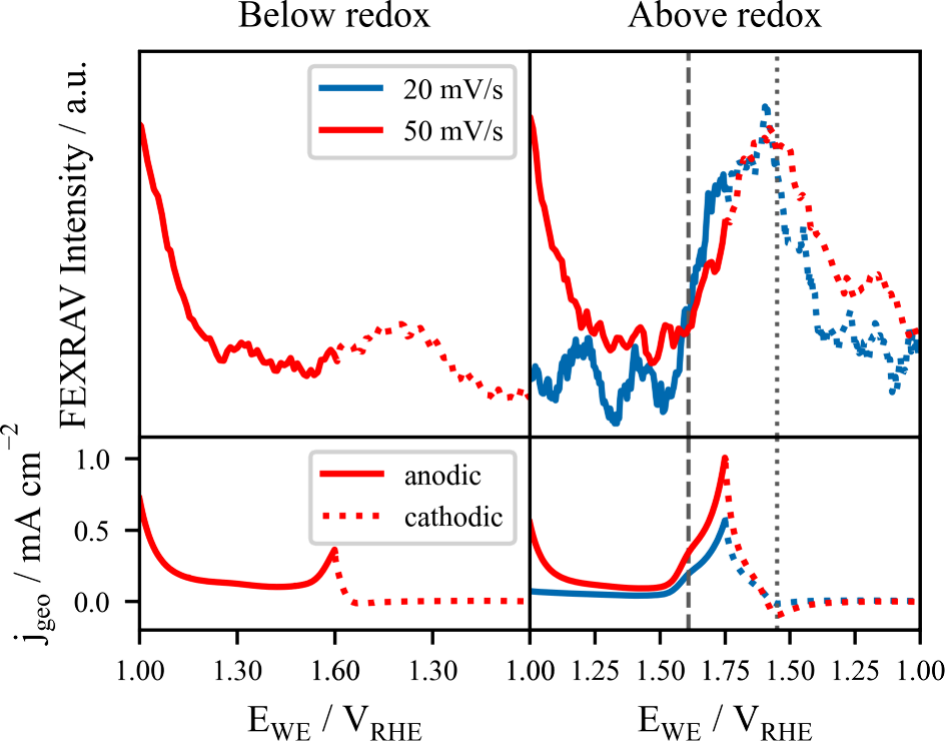
Potential-dependent study of the Cu-based electrocatalyst by FEXRAV at the characteristic excitation energy of the OER intermediate. (Top panel) FEXRAV signal at 932.6 eV, the spectral fingerprint of the intermediate species discussed above. Data is shown for two upper potential limits (UPLs): below (1.60 V_RHE_, left panel) and above (1.75 V_RHE_, right panel) the anodic redox feature. Also two scan rates are depicted: 20 mV/s (blue) and 50 mV/s (red). (Bottom panel) Corresponding CVs for the UPLs and scan rates mentioned, with the anodic branch (forward cycling direction) depicted as thick lines and the cathodic branch (backward potential sweep) as dashed lines. Gray dashed lines highlight the potentials of the redox couple in the anodic and cathodic branches.

To uncouple potentially undesired electrochemical reactions, FEXRAV measurements were performed at scan rates of 20 and 50 mV/s, as shown in Figure 2, with a slightly better signal quality due to the slower scanning (more points per mV). The 20 mV/s FEXRAV measurement shows a steep increase of the spectroscopic signal in the anodic branch around the anodic redox feature (∼1.62 V_RHE_). The smoother increase of the FEXRAV signal at 50 mV/s is due to the different scan rate, but the overall electrochemical behavior is independent of the scanning rate, indicating a system without diffusion-limited reactions (as further supported by the similarities between both CVs in the bottom panel of Figure 2).

In good agreement with the reversibility of the electrocatalyst observed with XAS before and after OER conditions, FEXRAV reveals that the original spectral fingerprint of the material is recovered in the backward potential sweep already at applied potentials below 1.3–1.4 V_RHE_ (depending on the scan rate), namely, below the cathodic part of the aforementioned redox couple. This finding further supports the relationship between the chemical transformations at the redox couple and the nature of the probed OER intermediate. While the FEXRAV analysis provides valuable insights into the redox and OER potential regions, it is important to note that a comprehensive understanding of the FEXRAV data for the full potential range shown in Figure 1b is beyond the scope of this work. The complete FEXRAV data set is provided in of the Supporting Information and involves multiple effects that are challenging to deconvolute at a single energy. These include potential Cu dissolution and the (dis)­appearance of specific Cu-containing species.

In conclusion, using *operando* Cu L_3_-edge XAS, we probed the chemical species of the key intermediate governing alkaline OER in a Cu-based electrocatalyst. The electronic structure of this metastable species resembles that of high-valent Cu complexes, especially those associated with the CuO_2_^–^ ion. The high-valent intermediate appears already at potentials as low as 1.62 V_RHE_, coinciding with the oxidative reaction typically attributed to the Cu^2+^ ↔ Cu^3+^ redox couple, while the original electrocatalyst state is recovered below 1.3–1.4 V_RHE_ depending on the scan rate of the cyclic voltammogram. This behavior suggests that the oxidative process at the redox potential is directly linked to the formation of the high-valent intermediate catalyzing the OER. Beyond the scope of the current work, further studies will explore the relationship between the probed OER intermediate, the limiting steps in the reaction’s pathway, and the phenomenon of transpassive Cu dissolution.

## Supplementary Material





## Notes

The Supporting Information includes a Materials and Experimental Methods section and a section with further ex-situ characterization of the Cu-based electrocatalyst as well as more extended details about the computed pDOS of the 7 Cu-based compounds, about the XAS measurements at OCP and about the FEXRAV measurements with different UPLs and scanning rates (PDF)
Transparent Peer Review report available (PDF)

## ⊥

Young Investigator Group Electrochemical Conversion of CO2. Helmholtz-Zentrum Berlin für Materialien und Energie GmbH, Berlin 14109, Germany
