# Supplementary material for: High-Valent Intermediate Observed in a Cu-Based OER Electrocatalyst by Operando X‑ray Absorption Spectroscopy
Source: J Phys Chem Lett. 2025 Jun 14;16(25):6328–33. doi: 10.1021/acs.jpclett.5c00944 (PMC12207661; doi:10.1021/acs.jpclett.5c00944)
Supplement: Supplementary file 1 [file jz5c00944_si_001.pdf]

Supplementary Information

High-valent intermediate observed in a Cu-based OER electrocatalyst by *operando* X-ray absorption spectroscopy

Raul Garcia-Diez,[*,†] Romualdus Enggar Wibowo,[†] Elmar Kataev,[†] Wilson Quevedo Garzon,[†] Marianne van der Merwe,[†] Daniel Duarte-Ruiz,[‡] Caterina Cocchi,[‡] and Marcus Bär[†,¶,§,‖]

[†] Interface Design, Helmholtz-Zentrum Berlin für Materialien und Energie GmbH (HZB), Berlin

[‡] Institute of Physics, Carl von Ossietzky Universität Oldenburg, Germany

[¶] Energy Materials In-Situ Laboratory Berlin (EMIL), HZB, Berlin, Germany

[§] Helmholtz Institute Erlangen-Nürnberg for Renewable Energy (HI ERN), Berlin, Germany

[‖] Dept. Chemistry and Pharmacy, Friedrich-Alexander-Universität Erlangen-Nürnberg (FAU), Germany

Materials and Experimental Methods

Electrode preparation and electrochemistry

The high surface area Cu-based electrocatalysts are prepared in a two-step process, including: chronoamperometric Cu electrodeposition in aq. 0.1 M $CuSO_4$ followed by the oxidation of the catalyst in aq. 1 M KOH to increase the electrochemically active surface area. First, a cuprous oxide Cu^{I}_2O is electrodeposited on a 15 nm thick Pt current collector coated either on a X-ray transparent 100 nm thick SiN window (for the synchrotron-based experiments) or on a SiO_x/Si wafer (for off-synchrotron characterization) using Chronoamperometry (CA) with an aq. 0.1 M $CuSO_4$ electrolyte, a Pt mesh as CE and a RHE RE, similar to the synthesis used in R. Garcia-Diez *et al*. (2025). Secondly, the high surface area of the electrocatalyst is obtained by anodization, i.e. applying 1.7 V_{RHE} potential in alkaline conditions (i.e. aq. 1M KOH), which triggers Cu dissolution and enables the characteristic shapes observed in **figure 1a**. Monitored *in-situ* by Cu L_3-edge XAS at the OÆSE endstation, this degradative process continues until the contribution to the spectroscopic signal of the electrochemically active oxides in the electrocatalyst's surface ($Cu^{II}(OH)_2$ and $Cu^{II}O$) dominates over the bulk species, the electrodeposited Cu^{I}_2O, as depicted exemplarily in **figure S1** below.

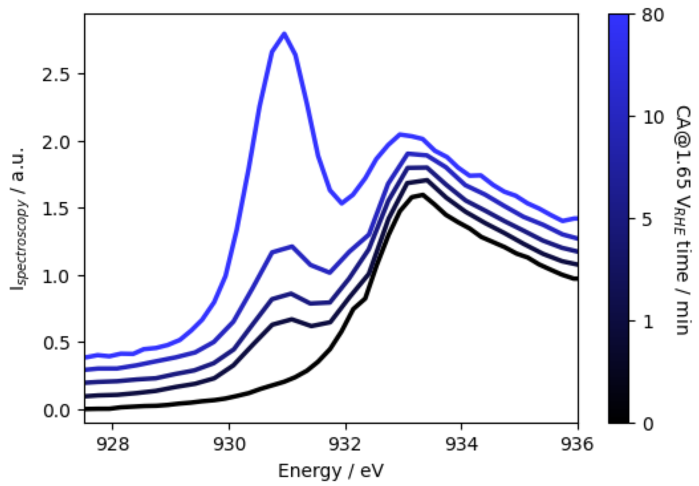

Figure S1: Example of the *in-situ* monitoring of the anodization of an electrodeposited cupper electrode: The 80 min oxidation of an *in-situ* electodeposited Cu catalyst in aq. 1 M KOH using CA@1.65 V_{RHE}, which increases the electrochemically active surface area based on the dissolution of Cu_2O (spectral fingerprint around 933.5 eV) at these high potentials, is monitored *in-situ* using Cu L_3-edge XAS. The spectroscopic signal evolves with CA time (color code) as contributions from electrochemically active oxides ($Cu(OH)_2$ and CuO) surpass those of the bulk-deposited species (Cu_2O).

In addition, the increase of the surface area of the Cu-based electrocatalyst at the aforementioned conditions has been electrochemically studied on a Cu electrode (250 um Cu foil) by applying consecutive 300 s potential holds at OER-relevant potentials (CA at 1.79 V_{RHE}) and measuring characterization Cyclic Voltammograms (CV) after each iteration, as shown in **figure S2**. The evolution of the electrocatalyst area with the cycle number can be evaluated by the current density at the maximum of the Cu(I)/Cu(II) anodic redox peak at 0.87 V_{RHE}, as depicted in the **bottom panel**. The electrochemically active surface area keeps growing as long as the potential hold is maintained at 1.79 V_{RHE} (number of cycles), likely related with an increase of the surface-to-bulk ratio, as complementarily observed by in-situ XAS (**figure S1**).

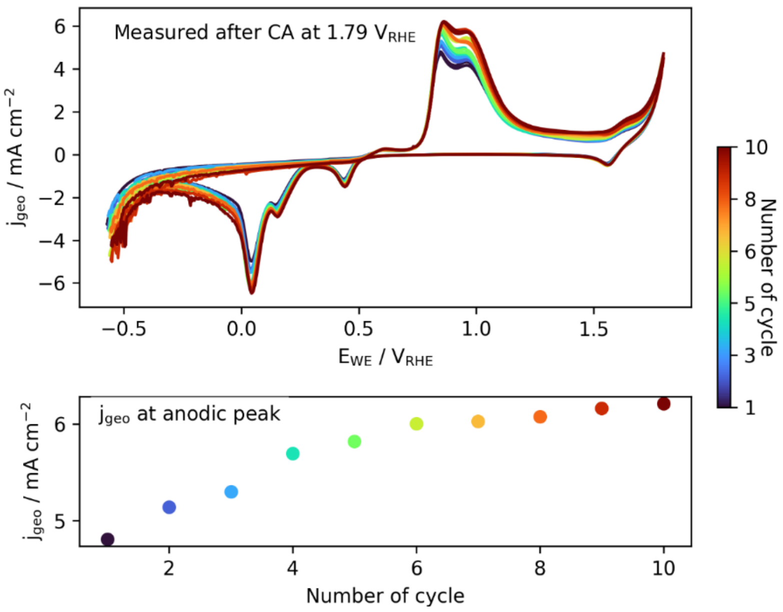

Figure S2. *Top panel:* Cyclic voltammograms (CVs) recorded in aq. 1 M KOH at 50 mV/s on a 250 µm Cu foil after consecutive 300 s potential holds at 1.79 V_{RHE}. CE: Pt mesh, RE: RHE. *Bottom panel:* Evolution of the anodic peak current density at ~0.87 V_{RHE} (Cu(I)/Cu(II) transition) as a function of the number of CA cycles. This redox feature serves as an electrochemical proxy for the active surface area, which increases progressively with each potential hold and suggests a continuous increase in the surface-to-bulk ratio.

The exact conditions of the electrochemical generation of the electrocatalysts used in this work are summarized in the following table:

	Electrodeposition (aq. 0.1M CuSO₄)		***Anodization (aq. 1M KOH)***				***Current collector***
**Catalyst**	E_{we} (V_{RHE})	Time (s)	CA at E_{we} (V_{RHE})	Time (min)	N_{cycles}	E_{we} range (V_{RHE})	
1a	-0.01*	100*	1.8*	80*	-	-	50nm Pt @Si wafer
1b	-0.01	100	1.8	80	-	-	15nm Pt @SiN window
1c	-0.01	100	-	-	5	-0.2 - 1.0	15nm Pt @SiN window
2	-0.06*	60*	1.75	33	-	-	15nm Pt @SiN window

Table S1: Electrochemical conditions used for the generation of the high surface area Cu-based electrocatalysts used in this work, labelled by the figure in which they are employed. Using a Pt wire as Counter Electrode (CE) and a Reversible Hydrogen Electrode (RHE) as Reference Electrode (RE), the copper electrodeposition is followed by an anodization which oxidizes the material and generates the characteristic high surface area found in these electrocatalysts. All electrochemical protocols were performed *in-situ* on a Pt-coated SiN window with the operando 3-electrode cell used for the XAS experiments, except those marked with an asterisk, which were done *ex-situ* and/or with a Pt-coated Si wafer as current collector.

Scanning Electron Microscopy (SEM)

The SEM data of the high surface area copper-based electrocatalyst, prepared with the same protocol as the initial electrocatalyst employed in the FEXRAV measurements (see Section about “Ex-situ characterization”), was obtained with 15 kV and a 530 000x magnification using a Phenom Pharos G2 Desktop FEG-SEM from Thermo Scientific. Energy-dispersive X-ray Spectroscopy SEM (EDX-SEM) was performed in map mode with 5 and 15 kV and the atomic concentrations were calculated with the PhenomWorld software.

X-ray Photoelectron Spectroscopy (XPS)

XPS measurements were performed with non-monochromatized Al K_α (1486.29 eV) X-ray source (SPECS XR 50, Germany) in the Energy Materials *In-Situ* Laboratory Berlin (EMIL) (Hsieh *et al.*, 2024). The photoelectrons were detected using a Scienta Omicron Argus CU electron analyzer (Sweden). The pass energy for the survey spectrum was 200 eV while for the Cu 2p core-level spectrum was set to 20 eV, resulting in an energy resolution of approximately (1.0 ± 0.3) eV.

Synchrotron-based experiments

Cu L_3-edge *operando* XAS and FEXRAV data were recorded in the OÆSE endstation of the Energy Materials In-Situ Laboratory Berlin (EMIL) at BESSY II, Helmholtz-Zentrum Berlin für Materialien und Energie GmbH. A planar grating monochromator with a density of 800 l mm^{-1} and a slit width of 100 μm was used for the measurements, leading to an energy resolution of ~250 meV at the Cu L_3-edge. The spectroscopic signal was collected in fluorescence mode at a photon-in/photon-out geometry using a 1.0 x 1.0 cm^2 Si photodiode optimized for X-ray photons (AXUV 100G, Optiodiode, USA) located at 45° to the surface. XAS scans were performed in continuous mode, with a read-out frequency of 10 Hz (same as in FEXRAV). Photon energy calibration of the Cu L_3-edge XAS was performed by employing the Si K-edge in 2nd order of the SiN window and Si wafer, as described in R. Garcia-Diez *et al*. (2025).

The electrochemical experiments were conducted using the three-electrode flow cell specifically designed for the OÆSE endstation, where a 1.0 x 0.5 mm^2 X-ray transparent SiN window with 100 nm thickness (Silson, UK) coated with 15 nm Pt is employed both as current collector for the electrocatalyst (as described above) and to separate the atmospheric pressure in the cell from the vacuum in the endstation and beamline (base pressure < 10^{-6} mbar).

Density Functional Theory

Density Functional Theory (DFT) calculations were performed using the plane-wave code Quantum Espresso (Giannozzi *et al.*, 2009 & 2017), with the Perdew-Burke-Ernzerhof (PBE, Perdew *et al.*, 1996) implementation of the generalized gradient approximation for the exchange-correlation potential. The following input structures were taken from the Materials Project database (Jain *et al.*, 2013): mp-505105 for orthorhombic $Cu^{II}(OH)_2$ (space group Cmc2(1), 4 Cu atoms and 8 O atoms per unit cell), mp-996956 for orthorhombic $Cu^{III}OOH$ (Pmn2(1), 2, 4), mp-997041 for orthorhombic $NaCu^{III}O_2$ (Cmcm, 4, 8), mp-3982 for orthorhombic $KCu^{III}O_2$ (Cmcm, 4, 8), mp-704645 for monoclinic $Cu^{II}O$ (C2/c, 1, 1), mp-361 for cubic Cu^{I}_2O (Pn-3m, 4, 2), and mp-20072 for trigonal $LaCu^{III}O_2$ (R-3m, 1, 2). The atomic positions were relaxed until the pressure was reduced to approximately 0 kbar. The following pseudopotentials (Hamann, 2013 and Schlipf & Gygi, 2015) were used to smooth out the core region of each atom: Cu_ONCV_PBE_sr.upf, O_ONCV_PBE_sr.upf, H_ONCV_PBE_sr.upf, K_ONCV_PBE_sr.upf, Na_ONCV_PBE_sr.upf, and La_ONCV_PBE_sr.upf. The Brillouin zone was sampled using a k-grid of 7x7x4 for CuO, 4x12x4 for $Cu(OH)_2$ and $NaCuO_2$, 4x8x4 for $KCuO_2$, 4x4x4 for $LaCuO_2$, 8x8x8 for Cu_2O and 4x4x8 for CuOOH. Cutoff values of 100 Ry for CuO and $LaCuO_2$, and 80 Ry for $KCuO_2$, CuOOH, $NaCuO_2$, Cu_2O and $Cu(OH)_2$ were chosen for the kinetic energy cutoff of the wave-functions, while for the kinetic energy cutoff of the charge density these values were magnified by a factor of 4 for CuO, Cu_2O and $LaCuO_2$, and 5 for $KCuO_2$, $NaCuO_2$, CuOOH, and $Cu(OH)_2$. Due to the ferromagnetic character of $Cu^{II}(OH)_2$, the pDOS of this compound is shown as the summation of the up and down states. For a better comparison with the X-ray Absorption Spectra and a consistent energy alignment of the pDOS, the Fermi

level is set in the center of the electronic band gap (or the alignment extracted from the Materials Project database for CuO), with energy offsets of 11.2, 6.15, 11.06, 5.4, 6.35, 10.5 and 8.05 eV for the Cu^{I}_2O, $Cu^{II}(OH)_2$, $Cu^{II}O$, $KCu^{III}O_2$, $NaCu^{III}O_2$, $LaCu^{III}O_2$, and $Cu^{III}OOH$ complexes, respectively. The $Cu^{III}_2O_3$ was not computed due to its well-known chemical instability, while the $Cu^{III}O_2^-$ ion simulation required a computational framework beyond the scope of this work.

Ex-situ characterization of the Cu-based electrocatalyst

For the *ex-situ* characterization, a high surface Cu-based electrocatalyst was prepared on a Pt-coated Si wafer by electrodepositing copper on the Pt current collector by 100 s of Chronoamperometry (CA) at -0.1 V_{RHE} in aq. 0.1M $CuSO_4$ followed by the oxidation of the film by an 80 min CA at 1.8 V_{RHE} in aq. 1M KOH, as displayed in **Figure S3a**. A Cyclic Voltammogram (CV) in 1M KOH with 50 mV/s scan rate is used to characterize the produced electrocatalyst, as shown in **Figure S3b.**

The ex-situ characterization of the electrocatalyst was performed after removing the electrode from the electrochemical medium and rinsing with DI water.

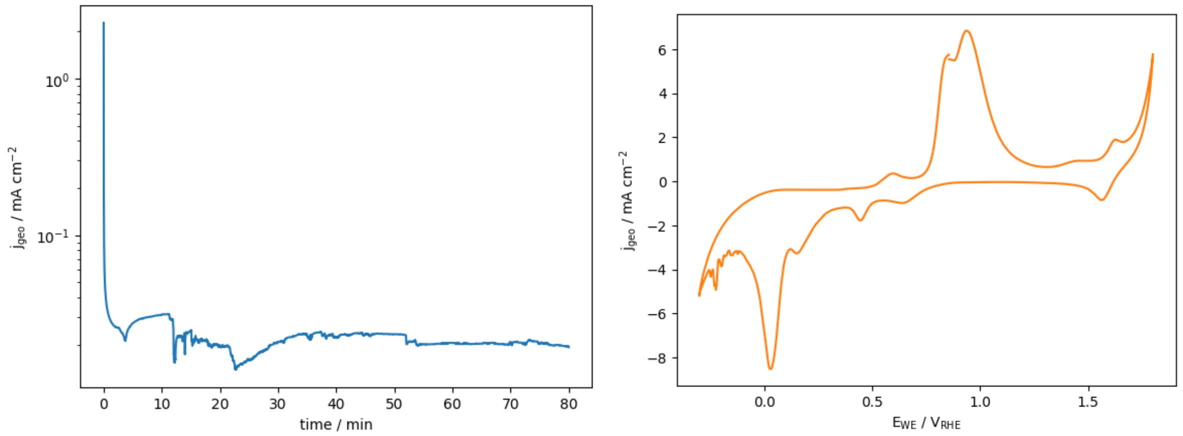

Figure S3: **a)** Current profile of the electrodeposited Cu thin film in aq. 1 M KOH using CA at 1.8 V_{RHE} during 80 min for the preparation of the high surface area Cu catalysts. **b)** CV of the high surface Cu-based electrocatalyst used for the ex-situ characterization. CV were recorded in aq. 1M KOH with the scan rate of 50 mV/s. Pt mesh was used as CE, while a RHE was used as reference electrode.

X-ray Photolectron Spectroscopy

Ex-situ surface analysis of the material was carried out by Al K_a XPS. In **Figure S4a**, a survey of the electrode is shown, where the characteristic peaks of copper and oxygen can be observed, associated with the electrocatalyst. The peaks of potassium (K) arise from remaining traces of the electrolyte (aq. 1M KOH) on the electrode are also detected (see SEM-EDX for further details), as well as the signal from the Pt current collector.

In the detailed core-level spectrum of Cu 2p depicted in **Figure S4b**, the characteristic strong satellite peaks of Cu^{2+} are clearly visible, as well as the chemical shift associated with this oxidation state (Cu foil as reference). These observations further support the findings by XAS that the species present at the surface of the electrocatalyst are of Cu(II) nature, likely $CuO/Cu(OH)_2$.

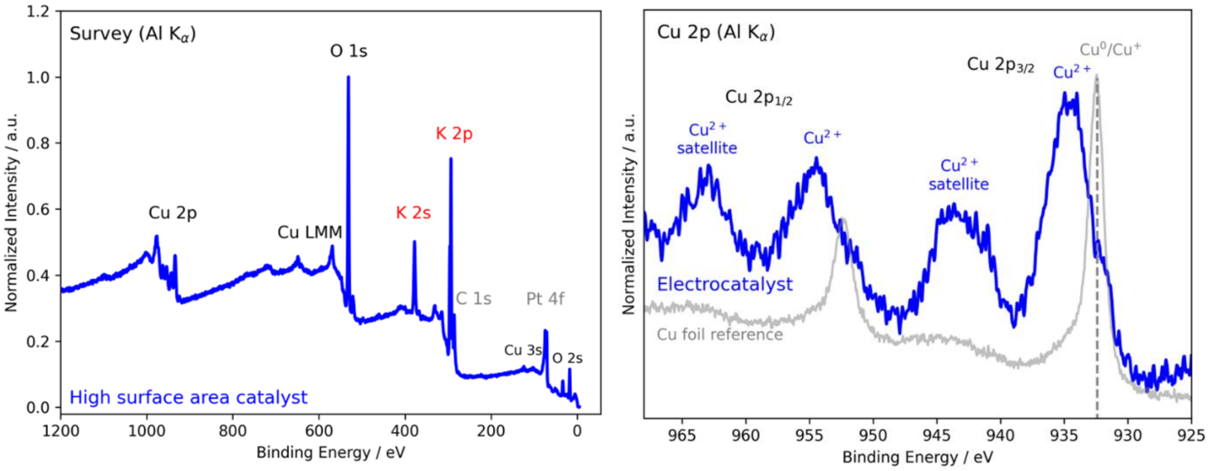

Figure S4: **a)** Survey and **b)** Cu 2p core-level spectra of the high surface Cu-based electrocatalyst. For comparison purposes, the spectrum of a Cu foil has been included in the Cu 2p spectrum.

SEM and EDX-SEM

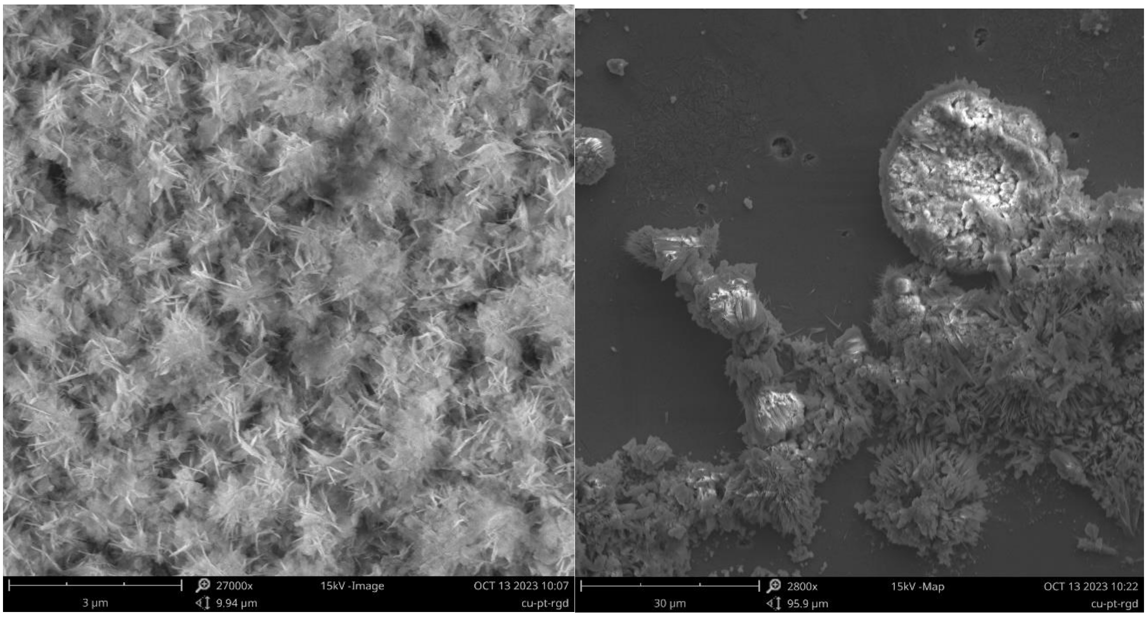

Figure S5: Low magnification 15 kV SEM images of the high surface area Cu-based electrocatalyst

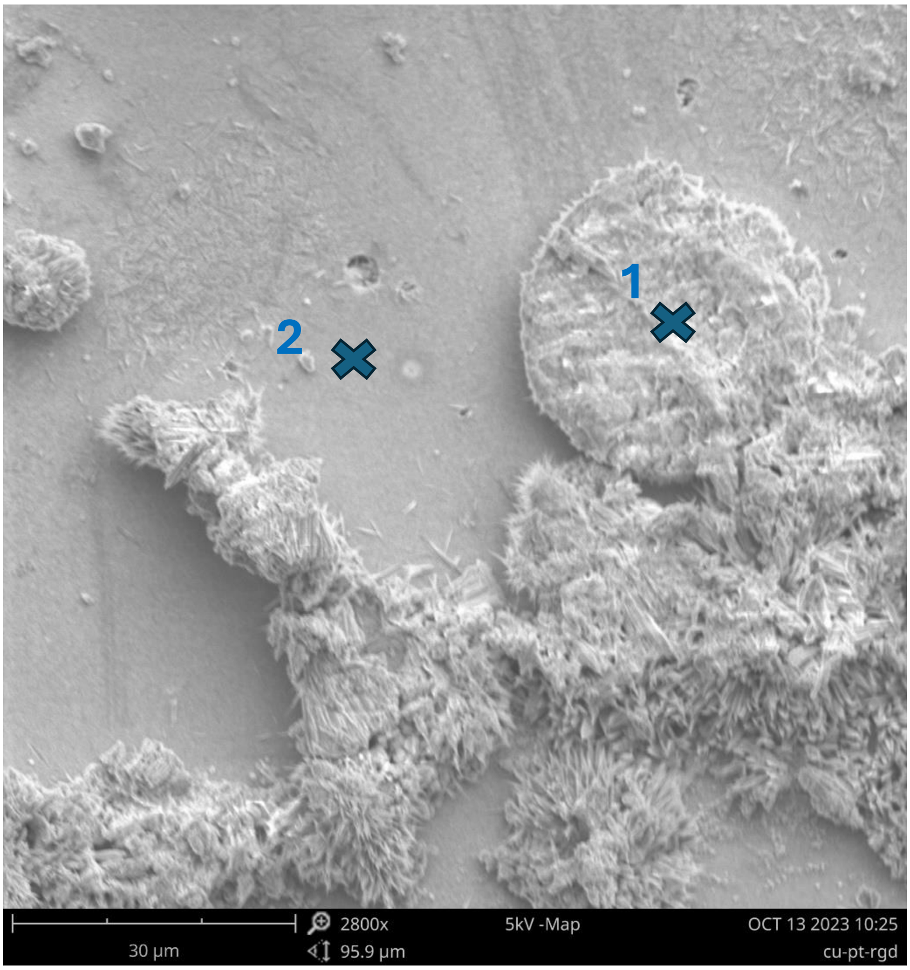

Figure S6: 5 kV SEM image used for the EDX analysis of the high surface area Cu-based electrocatalyst. The 2 analyzed sample spots are shown as crosses.

Element	Spot 1 (5 kV)	Spot 1 (15 kV)	Spot 2 (5 kV)	Spot 2 (15 kV)
Cu	56.14	71.41	4.33	7.05
O	43.57	17.61	8.24	6.33
K	-	10.27	79.15	1.62
Pt	0.30	-	6.60	36.03
Si	-	0.72	1.68	48.96

Table S2: SEM-EDX at 5 and 15 kV: Elemental analysis of 2 sample spots of the electrode, corresponding to: 1) areas with high concentration of the high surface area Cu-based electrocatalyst and 2) less covered areas. The atomic concentrations are shown for all the elements of the electrode.

Considerations about XAS at OCP

In **figure 1c**, XAS during Chrono-Amperometry (CA) at 1.75 V_{RHE} and at Open Circuit Potential (OCP) conditions (before and after CA) are shown. Understanding the behaviour of the catalyst during the post-CA OCP is important to correlate the probed chemical species with the electrochemical response presented. Therefore, the relaxation of the OCP after the exposition of the catalyst to high anodic potentials during the potential hold is investigated here on a Cu

electrode (250 um Cu foil), mirroring the electrochemical protocol used in our operando XAS study.

Figure S7 bottom shows the temporal evolution of OCP after a 300 s potential hold at 1.79 V_{RHE} (current density during CA shown in the **top panel**). It can be observed how OCP consistently relaxes back to its original value of approximately 0.75 V_{RHE} after the applied potential is removed. OCP decays close to the initial OCP value within ~60 seconds and fully returns to the baseline (~0.75 V_{RHE}) within 120 seconds. Since our operando XAS measurements at OCP were taken after a 60 seconds relaxation period (and each spectrum acquisition also takes approximately 60 seconds), the spectra recorded at OCP reliably represent the material at conditions similar to its initial OCP state.

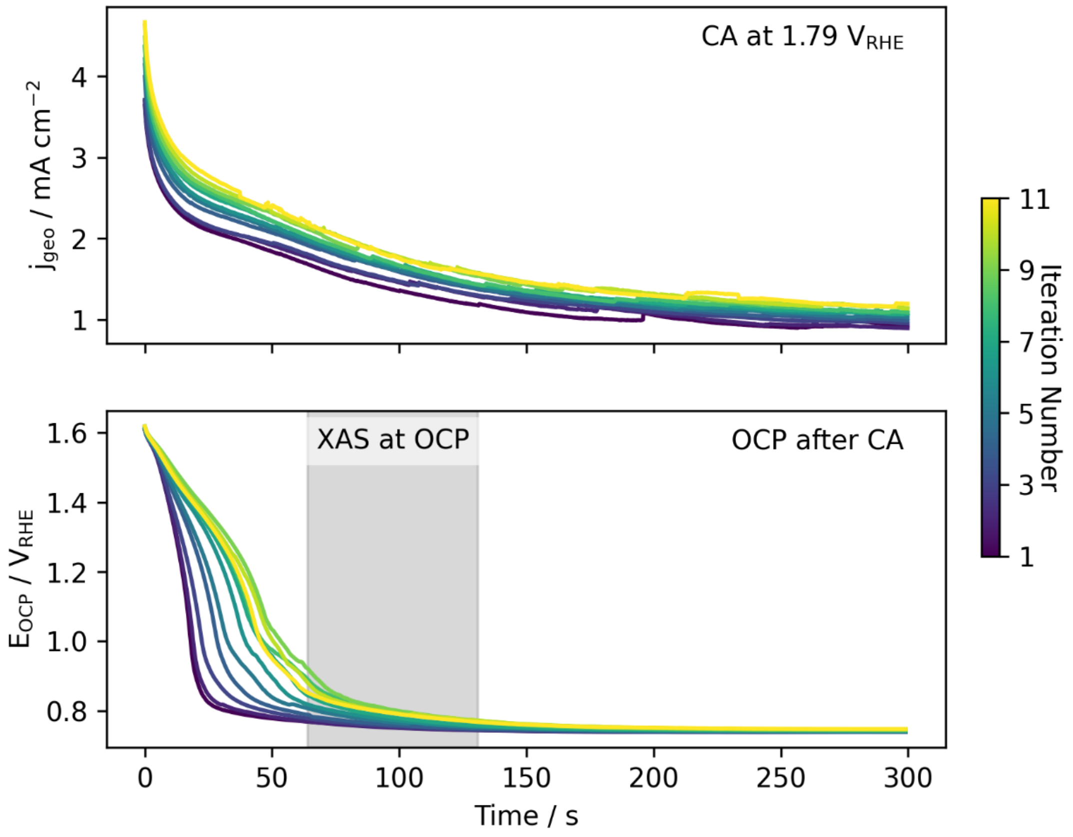

Figure S7: *Top panel:* Chronoamperometric (CA) current response during a 300 s potential hold at 1.79 V_{RHE}, showing the current density evolution under OER conditions. *Bottom panel:* Temporal evolution of the open circuit potential (OCP) following the CA step. After the applied potential is removed, the OCP decays toward its initial value of ~0.75 V_{RHE} within ~60 seconds and fully recovers its original value after ~120 seconds. For completeness, the period in which the XAS at OCP is measured is highlighted in gray.

Dissolution of copper from the electrocatalyst

The loss of the Cu-associated spectroscopic signal at the electrode, observed in the spectro-micrograph of the electrocatalyst after the experiment, confirms Cu dissolution from the electrocatalyst at the high potentials related to OER (>1.62 V_{RHE}).

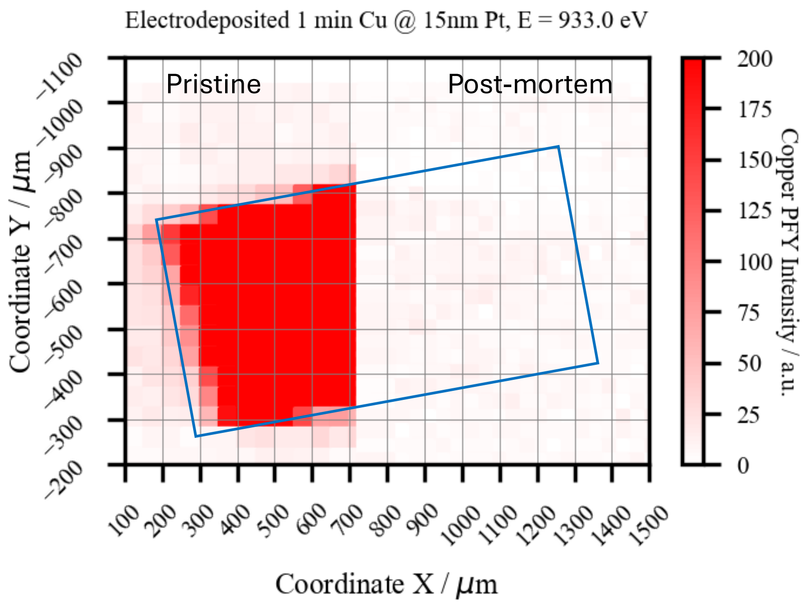

Figure S8: Spectro-micrograph of the electrocatalyst before (left half side) and after OER conditions (right half-side) obtained with a Cu L_3-edge resonant excitation energy of 933.0 eV collected in Cu Lα-PFY mode. The lack of Cu-containing material after several CA protocols at applied potentials higher than 1.62 VRHE is related with the severe transpassive dissolution of Cu at high OER-relevant potentials. The contour of the 1000 × 500 μm^2 SiN membrane within the Si frame (with a 15° tilt) is depicted in blue.

Complete set of pDOS of 7 Cu-based compounds with relevant oxidation states

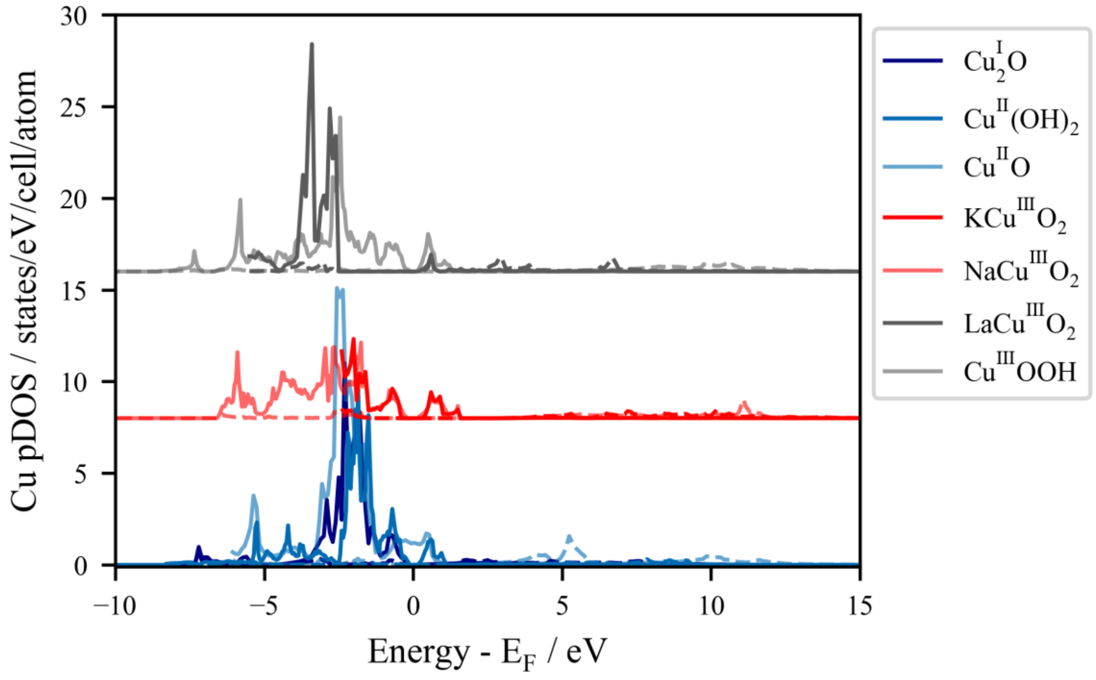

Figure S9: Calculated Cu d- and s-states (thick and dashed lines, respectively) shown in a wider energy range than in figure 1d: Projected density of states (pDOS) calculated by *ab initio* Density Functional Theory (DFT) of 7 Cu-containing complexes potentially related to the electrocatalyst species.

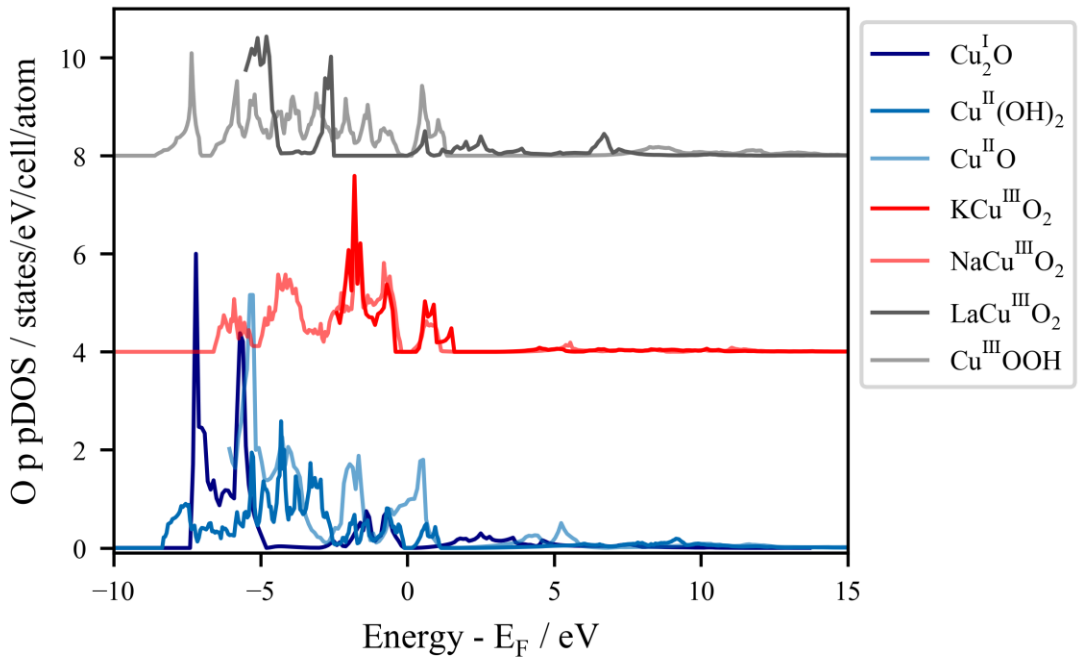

Figure S10: Calculated O p-states shown in a wider energy range than in figure 1d: Projected density of states (pDOS) calculated by *ab initio* Density Functional Theory (DFT) of 7 Cu-containing complexes potentially related to the electrocatalyst species.

FEXRAV with different UPLs and scanning rates

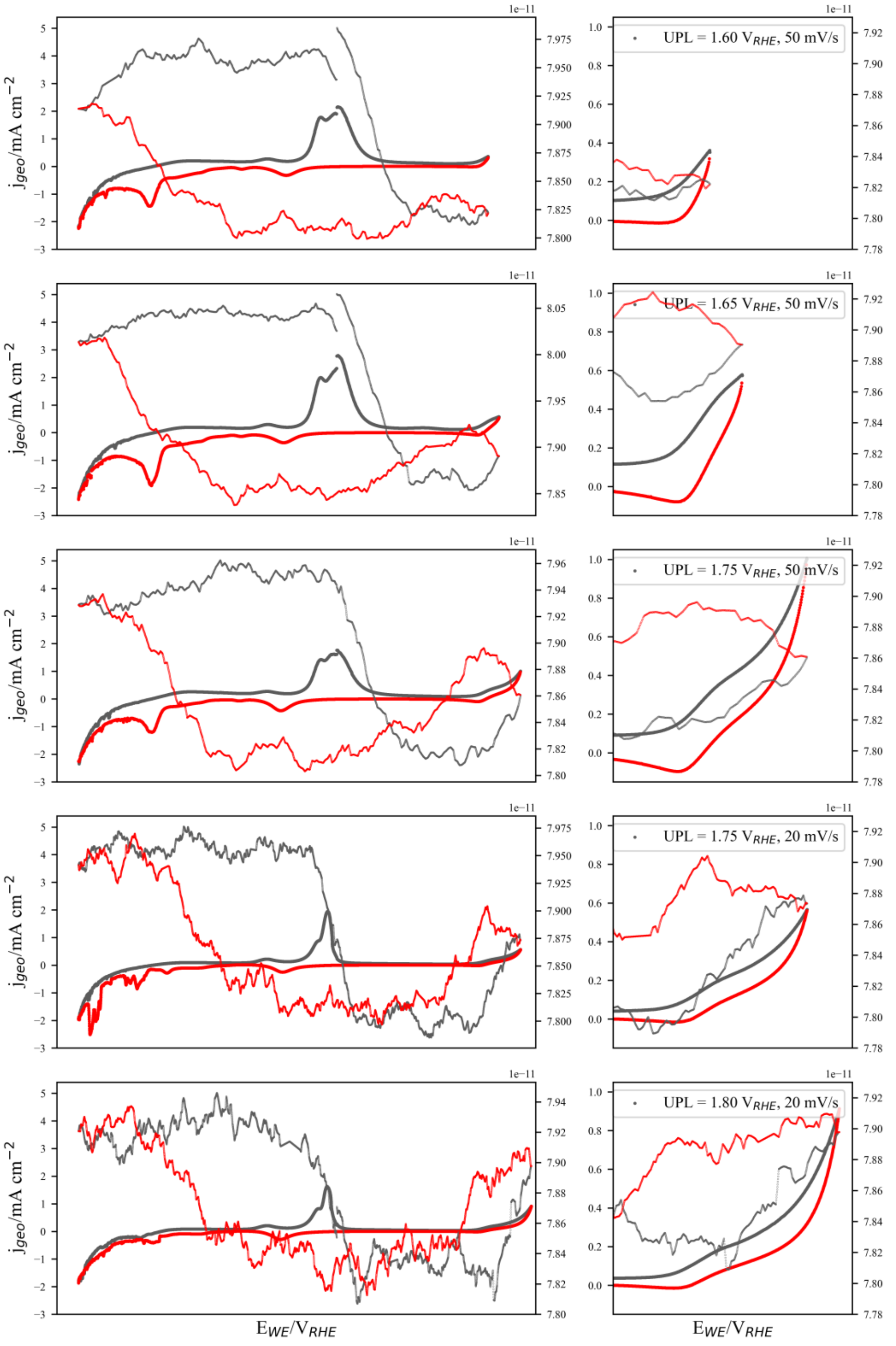

Figure S11: FEXRAV signal measured at the excitation energy of 932.6 eV for different Upper Potential Limits (UPLs) and scan rates using the electrocatalyst shown in **figure 2** of the main text. Bolded line represents the CV profile of the catalysts, while thinner line corresponds to the FEXRAV intensity. The left panel depicts the whole potential range of the Cyclic Voltammogram (CV), where oxidation, dissolution and other effects can be observed, and the right panel shows the potential window of interest for the scientific scope of this work, redox couple region and OER-relevant potentials.

Literature

Garcia-Diez, R.; Frisch, J.; van der Merwe, M.; Wibowo, R.E.; Gorgoi, M.; Kataev, E.; Jimenez, C.E., Arce, M.D.; Smith, W.; Quevedo-Garzon, W.; Wilks, R.G.; Wallacher, D., Reinschlüssel, L.J.; Tok, G.C.; Gasteiger, H.A.; Bär, M., **2025**.The OÆSE endstation at BESSY II: operando X-ray absorption spectroscopy for energy materials. Journal of Synchrotron Radiation, 32

Giannozzi, P., Andreussi, O., Brumme, T., Bunau, O., Nardelli, M.B., Calandra, M., Car, R., Cavazzoni, C., Ceresoli, D., Cococcioni, M. and Colonna, N., **2017**. Advanced capabilities for materials modelling with Quantum ESPRESSO. Journal of physics: Condensed matter, 29(46), p.465901.

Giannozzi, P., Baroni, S., Bonini, N., Calandra, M., Car, R., Cavazzoni, C., Ceresoli, D., Chiarotti, G.L., Cococcioni, M., Dabo, I. and Dal Corso, A., **2009**. QUANTUM ESPRESSO: a modular and open-source software project for quantum simulations of materials. Journal of physics: Condensed matter, 21(39), p.395502.

Hamann, D.R., **2013**. Optimized norm-conserving Vanderbilt pseudopotentials. Physical Review B—Condensed Matter and Materials Physics, 88(8), p.085117.

Hsieh, T.-E., Frisch, J., Wilks, R. G., Papp, C., Bär, M., **2024**. Impact of Catalysis-Relevant Oxidation and Annealing Treatments on Nanostructured GaRh Alloys. ACS Appl. Mater. Interfaces, *16* (15), p.19858.

Jain, A., Ong, S.P., Hautier, G., Chen, W., Richards, W.D., Dacek, S., Cholia, S., Gunter, D., Skinner, D., Ceder, G. and Persson, K.A., **2013**. Commentary: The Materials Project: A materials genome approach to accelerating materials innovation. APL materials, 1(1).

Perdew, J.P., Burke, K. and Ernzerhof, M., **1996**. Generalized gradient approximation made simple. Physical review letters, 77(18), p.3865.

Schlipf, M. and Gygi, F., **2015**. Optimization algorithm for the generation of ONCV pseudopotentials. Computer Physics Communications, 196, pp.36-44.
