# Supplementary material for: High-Valent Intermediate Observed in a Cu-Based OER Electrocatalyst by Operando X‑ray Absorption Spectroscopy
Source: J Phys Chem Lett. 2025 Jun 14;16(25):6328–33. doi: 10.1021/acs.jpclett.5c00944 (PMC12207661; doi:10.1021/acs.jpclett.5c00944)
Supplement: Supplementary file 2 [file jz5c00944_si_002.pdf]

The Journal of Physical Chemistry Letters

Department of Interface Design

Prof. Dr. Marcus Bär
Dr. Raul Garcia-Diez
Albert-Einstein-Str. 15

12489 Berlin, Germany

Tel +49 30 8062-15640

***marcus.baer**@helmholtz-berlin.de
***raul.garcia_diez**@helmholtz-berlin.de

Berlin, 21.05.2025

Resubmission of manuscript No. jz-2025-00944c to J. Phys. Chem. Letters

Dear Editor,

Thank you for the opportunity to address the reviewers' comments and revise the manuscript accordingly. Please find attached the revised version of our manuscript (with and without editing marks) along with our accompanying responses to the each of the referees' concerns in the following below. All comments by the referees are shown by "**bold text**", and all revisions in the manuscript are marked with red text. In addition, all the non-scientific requests made by the editorial office have been addressed i.e. TOC figure in tiff format, complete address of the affiliations and correction of some citations.

We would be happy if the co-author's name **Quevedo Garzon, Wilson** could be written without hyphen (as in the original manuscript). We apologize in advance for the inconvenience.

The reviewers' comments were highly insightful, and greatly helped us to improve the quality of our manuscript. We hope the revisions to the manuscript and accompanying responses will be sufficient to make our manuscript suitable for publication in The Journal of Physical Chemistry Letters.

With best regards,
Corresponding Authors

Prof. Dr. Marcus Bär
Head of the Dept. Interface Design

Dr. Raul Garcia-Diez
Senior Scientist

Department of Interface Design
Helmholtz Zentrum Berlin für Materialen und Energie GmbH
Tel: (+49) 30 8062 – 15640 / 15641
emails: ***marcus.baer**@helmholtz-berlin.de, ***raul.garcia_diez**@helmholtz-berlin.de,

Helmholtz-Zentrum Berlin für Materialien und Energie GmbH | Hahn-Meitner-Platz 1 | 14109 Berlin

Response to Reviewers' Comments

Journal: The Journal of Physical Chemistry Letters
Manuscript ID: jz-2025-00944c
Manuscript Title: " High-valent intermediate observed in a Cu-based OER electrocatalyst by *operando* X-ray absorption spectroscopy "
Manuscript Authors: Raul Garcia-Diez, Romualdus Enggar Wibowo, Elmar Kataev, Wilson Quevedo Garzon, Marianne van der Merwe, Daniel Duarte-Ruiz, Caterina Cocchi, and Marcus Bär

Reviewer: 1

Comments:

In the submitted manuscript, the authors characterize electrodeposited Cu-based electrocatalysts for alkaline OER with operando XAS measurements. Interestingly, they detect the emergence of a highly oxidized Cu species in an oxidation process at 1.62V, which they assign to a CuO2- species. While the authors present interesting results, the work needs major revisions to be considered for publishing, especially regarding the interpretation that the transient species is related to the OER mechanism.

Why should the highly oxidized species be related to the OER activity and not be the oxidized Copper species that leads to dissolution? I don't see any evidence in the presented data that it participates in the OER. All data show that the intensity of the signal at 932.6 eV peaks at the Cu oxidation peak around 1.6 V and then decreases, while the current increases likely related to OER. How can the species be related to the OER mechanism if it decreases significantly when the oxidative potential is still increased?

Thank you very much, we appreciate your insightful question as it helps us to strengthen the clarity and presentation of our results. Specifically, we agree that the original visualization of the FEXRAV data in Figure 2 may have led to some confusion regarding the relationship between the abundance of the high valent Cu species and the potential region in which it increases. In fact, the abundance of the species increases above 1.62 V_{RHE} in the forward scan direction (anodic redox feature) until reaching a maximum already in the reverse potential sweep at 1.55 V_{RHE} (cathodic redox), where the

trend changes and the intensity starts decreasing since it is outside the oxidative potential window related to this redox transition.

To address this, we have revised Figure 2 to more clearly illustrate this effect. A new figure has been created, and the surrounding text in the manuscript has been accordingly modified to reflect these changes and better guide the reader through our interpretation.

Changes in the manuscript (p. 9-10 and figure 2) in red:

The FEXRAV signal measured at 932.6 eV is shown in the top panel of figure 2 while the bottom panel depicts the corresponding CVs. Collected for two different UPLs, the FEXRAV data reveals that the intermediate-related spectral fingerprint only appears for positive enough potentials, specifically, when the UPL is above the redox transition (see figure S11 of SI for more UPLs and scanning rates), the same potential regime where OER occurs. When looking in more detail at the FEXRAV signal, it is evident that the spectroscopic intensity related to the new species, i.e. the abundance of the chemical species, grows for applied potentials above 1.62 V_{RHE} in the forward cycling direction (redox feature in the anodic branch, highlighted with a grey dashed line in figure 2) and continues growing for higher potentials in the OER region until reaching a maximum at 1.55 V_{RHE} already in the backward direction (redox feature in the cathodic branch, grey dashed line), where the trend is reversed. At lower potentials in the reverse potential sweep, the abundance of this new species begins to decrease until its original concentration is reached around 1.0 V_{RHE}. The increase of the FEXRAV signal, which only occurs at the potential window between the anodic and cathodic features of this redox transition, which lies in the potential regime of OER; suggests that the oxidation process and chemical transformation occurring at this redox potential are directly linked to the formation of the OER intermediate. Importantly, there is no evidence that the oxidative process at the redox feature produces a distinct product or intermediate compared to what is observed under OER conditions. Instead, the results align with prior findings in this study indicating that the OER intermediate likely corresponds to an oxidized species with a high-valent Cu nature.

To uncouple potentially undesired electrochemical reactions, FEXRAV measurements were performed at scan rates of 20 mV/s and 50 mV/s, as shown in figure 2, with a slightly better signal quality due to the slower scanning (more points per mV). The 20 mV/s FEXRAV measurement shows a steep increase of the spectroscopic signal in the anodic branch around the anodic redox feature (~ 1.62 V_{RHE}).

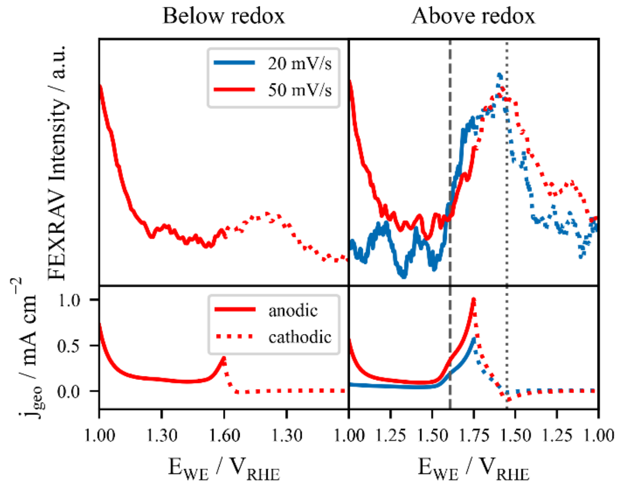

Figure 2: Potential-dependent study of the Cu-based electrocatalyst by FEXRAV at the characteristic excitation energy of the OER intermediate. **Top panel)** FEXRAV signal at 932.6 eV, the spectral fingerprint of the intermediate species discussed above. Data is shown for two upper potential limits (UPLs): below (1.60 V_{RHE}, left panel) and above (1.75 V_{RHE}, right panel) the anodic redox feature. Also two scan rates are depicted: 20 mV/s (blue) and 50 mV/s (red). **Bottom panel)** Corresponding CVs for the UPLs and scan rates mentioned, with the anodic branch (forward cycling direction) depicted as thick lines and the cathodic branch (reverse potential sweep) as dashed lines. Gray dashed lines highlight the potentials of the redox couple in the anodic and cathodic branches.

It would be important to show the Cu dissolution as a function of potential and compare it to the spectroscopic data. If the species is indeed related to the dissolution, this finding would be important for the fundamental understanding of the electrochemical (in)stability of Cu, which can be argued to be of much higher general interest than OER on Cu. So, to clearly resolve this ambiguity would increase the importance of the manuscript.

Thank you for your valuable comment. We agree that the dissolution behaviour is an important aspect deserving further exploration. This topic is currently being investigated in our ongoing work, and will be addressed in detail in a forthcoming, more extensive manuscript dedicated specifically to dissolution studies.

(…)

In the main text we have included in p. 11: "Beyond the scope of the current work, further studies will explore the relationship between the probed OER intermediate, the limiting steps in the reaction's pathway and the phenomenon of transpassive Cu dissolution."

It is unclear what the authors mean with an OER intermediate throughout the manuscript. This would conventionally be an intermediate of oxygen adsorbed on the catalyst surface. The authors should be more clear in the description of the OER mechanism that they want to elucidate.

We thank the reviewer for this important observation. We agree that the term "OER intermediate" can be ambiguous, and we have revised the manuscript to clarify our intended meaning. In our study, we refer to that intermediate present in the OER mechanism pathway as a species that accumulates on the catalyst surface within the timescale accessible by our measurements.

Such intermediates are typically associated with the rate-determining step of the OER mechanism, as only these can reach detectable concentrations under reaction conditions. Therefore, our measurements allow us to probe only those intermediates that are kinetically stabilized and accumulate during the reaction, rather than all transient species involved in the full OER pathway. The text has been updated accordingly to more precisely convey this definition and the mechanistic interpretation behind our approach.

Changes in the manuscript (p.3) in red:

To resolve these ambiguities, operando X-ray absorption spectroscopy (XAS) emerges as an ideal tool for probing intermediates that may otherwise evade detection due to their short lifetimes. The accumulation of an intermediate within the timescale accessible by *operando* methods requires that the OER intermediate stabilizes kinetically on the surface of the catalyst and becomes dominant, typically associated with the species at the rate-determining step in the mechanism pathway [21]. In particular, XAS at the Cu $L_{2,3}$-edge provides exceptional chemical sensitivity and insights into electronic transitions involving the active 3d-state.

The authors write, "CVs show a slight dependence on the sample's history and the solution's molarity, but the overall shape remains consistent". These differences are, however, not shown in the manuscript. Based on the experimental section, it appears that several different methods were used to prepare the samples. It is unclear which sample was prepared exactly how and how comparable these different samples are. A list/table would help. The sample characterization of all differently prepared samples, at least in the form of CVs, is needed.

We thank the reviewer for this constructive suggestion. To improve clarity and traceability, we have added a table in the *"Electrode Preparation and electrochemistry"* section of the Supporting Information o better document the preparation conditions detailing the specific synthesis parameters used for each of the high surface area Cu-based electrocatalysts employed throughout the study. This table provides a clear overview of the different preparation methods and ensures the comparability of the samples used in the various experiments. Additionally, we have included two new figures in the Supporting Information (Fig. S2 and S3) that illustrate the characteristic electrochemical behavior during the catalyst preparation by potential hold.

Moreover, the observed variations in the CVs arising from the electrode history primarily affect the redox features associated with the Cu(I)/Cu(II) transition, as observed in the new figure S2. However, the potential window of interest for this study—above 1.6 V, corresponding to the Cu(II)/Cu(III) redox process—remains consistent across different sample histories. As a result, the main conclusions drawn in our manuscript are robust and independent of the exact preparation conditions of the high surface area catalyst.

Changes and additions in SI (pp.2-3) in red:

In addition, the increase of the surface area of the Cu-based electrocatalyst at the aforementioned conditions has been electrochemically studied on a Cu electrode (250 um Cu foil) by applying consecutive 300 s potential holds at OER-relevant potentials (CA at 1.79 V_{RHE}) and measuring characterization Cyclic Voltammograms (CV) after each iteration, as shown in **figure S2**. The evolution of the electrocatalyst area with the cycle number can be evaluated by the current density at

the maximum of the Cu(I)/Cu(II) anodic redox peak at 0.87 V_{RHE}, as depicted in the **bottom panel**. The electrochemically active surface area keeps growing as long as the potential hold is maintained at 1.79 V_{RHE} (number of cycles), likely related with an increase of the surface-to-bulk ratio, as complementarily observed by in-situ XAS (**figure S1**).

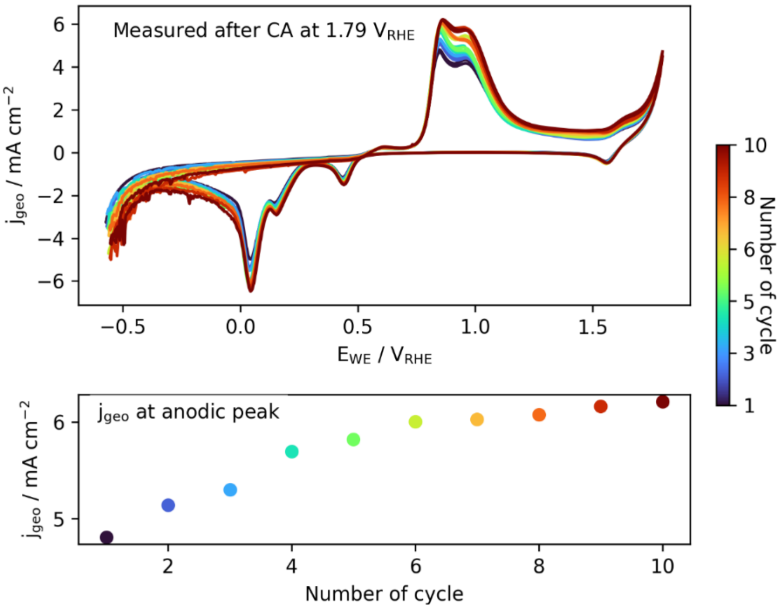

Figure S2. *Top panel:* Cyclic voltammograms (CVs) recorded in aq. 1M KOH at 50 mV/s on a 250 µm Cu foil after consecutive 300 s potential holds at 1.79 V_{RHE}. CE: Pt mesh, RE: RHE. *Bottom panel:* Evolution of the anodic peak current density at ~0.87 V_{RHE} (Cu(I)/Cu(II) transition) as a function of the number of CA cycles. This redox feature serves as an electrochemical proxy for the active surface area, which increases progressively with each potential hold and suggests a continuous increase in the surface-to-bulk ratio.

The exact conditions of the electrochemical generation of the electrocatalysts used in this work are summarized in the following table:

	Electrodeposition (aq. 0.1M $CuSO_4$)		***Anodization (aq. 1M KOH)***				***Current collector***
**Catalyst**	E_{we} (V_{RHE})	Time (s)	CA at E_{we} (V_{RHE})	Time (min)	N_{cycles}	E_{we} range (V_{RHE})	
1a	-0.01*	100*	1.8*	80*	-	-	50nm Pt @Si wafer
1b	-0.01	100	1.8	80	-	-	15nm Pt @SiN window
1c	-0.01	100	-	-	5	-0.2 - 1.0	15nm Pt @SiN window
2	-0.06*	60*	1.75	33	-	-	15nm Pt @SiN window

Table S1: Electrochemical conditions used for the generation of the high surface area Cu-based electrocatalysts used in this work, labelled by the figure in which they are employed. Using a Pt wire as Counter Electrode (CE) and a Reversible Hydrogen Electrode as Reference Electrode (RHE RE), the copper electrodeposition is followed by an anodization which oxidizes the material and generates the characteristic high surface area found in these electrocatalysts. All electrochemical protocols were performed *in-situ* on a Pt-coated SiN window with the operando 3-electrode cell used for the XAS experiments, except those marked with an asterisk, which were done *ex-situ* and/or with a Pt-coated Si wafer as current collector.

Additions in SI (pp.5) in red:

For the *ex-situ* characterization, a high surface Cu-based electrocatalyst was prepared on a Pt-coated Si wafer by electrodepositing copper on the Pt current collector by 100 s of Chronoamperometry (CA) at -0.1 V_{RHE} in aq. 0.1M $CuSO_4$ followed by the oxidation of the film by an 80 min CA at 1.8

V_{RHE} in aq. 1M KOH, as displayed in **Figure S3a**. A Cyclic Voltammogram (CV) in 1M KOH with 50 mV/s scan rate is used to characterize the produced electrocatalyst, as shown in **Figure S3b.**

The ex-situ characterization of the electrocatalyst was performed after removing the electrode from the electrochemical medium and rinsing with DI water.

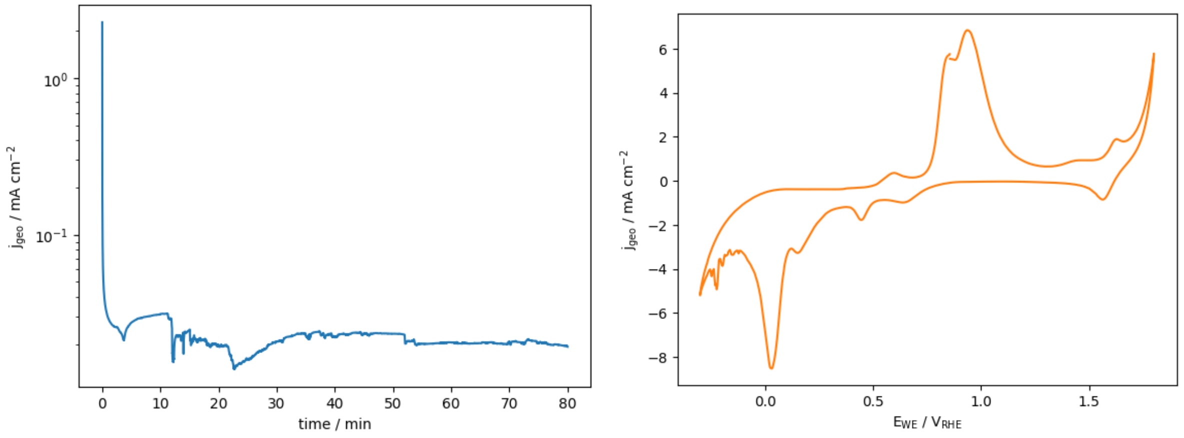

Figure S3: **a)** Current profile of the electrodeposited Cu thin film in aq. 1 M KOH using CA at 1.8 V_{RHE} during 80 min for the preparation of the high surface area Cu catalysts. **b)** CV of the high surface Cu-based electrocatalyst used for the ex-situ characterization. CV were recorded in aq. 1M KOH with the scan rate of 50 mV/s. Pt mesh was used as CE, while a RHE was used as reference electrode.

The authors point out that the spectral feature before and after the CA are the same at OCP, even though the potential has significantly shifted to higher potentials, indicating a more oxidized catalyst. How can this be explained?

We thank the reviewer for this important question. To clarify, although the catalyst is exposed to high anodic potentials during the chronopotentiometric holds, the open-circuit potential (OCP) consistently relaxes back to its original value of approximately 0.75 V_{RHE} over time after the applied potential above the Cu(II)/Cu(III) redox feature is removed.

To further investigate this behavior, we performed a dedicated experiment that mirrors the electrochemical protocol used in our operando XAS study. In this experiment, we monitored the OCP following a hold at OER-relevant potentials (1.75 V_{RHE}) and observed that it decays close to the initial OCP value within ~60 seconds, and fully returns to the baseline (~0.75 V) within 120 seconds. Since our operando XAS measurements at OCP were taken after a 60-second relaxation period (and each spectrum acquisition also takes approximately 60 seconds), we are confident that the spectra recorded at OCP reliably represent the material in its relaxed, resting state. This study has been added to the SI in the section "Considerations about XAS at OCP".

We acknowledge that referring to the "initial OCP" as a quantitative reference in Figure 1b may have caused confusion, and we apologize for this. We have clarified this point in the revised manuscript and ensured that the labeling in Figure 1b no longer implies a significant shift in the catalyst state at OCP after CA.

Changes in the manuscript (p.6) in red:

Notably, this new feature disappears when returning to a relaxed state (OCP after CA. See Considerations about XAS at OCP in Supplementary Information for more details about the relaxation behavior of the electrocatalyst). In fact, the spectrum recorded at OCP after OER coincides with the data collected at pristine OCP (…)

Edit in figure 1, panel B (p.5):

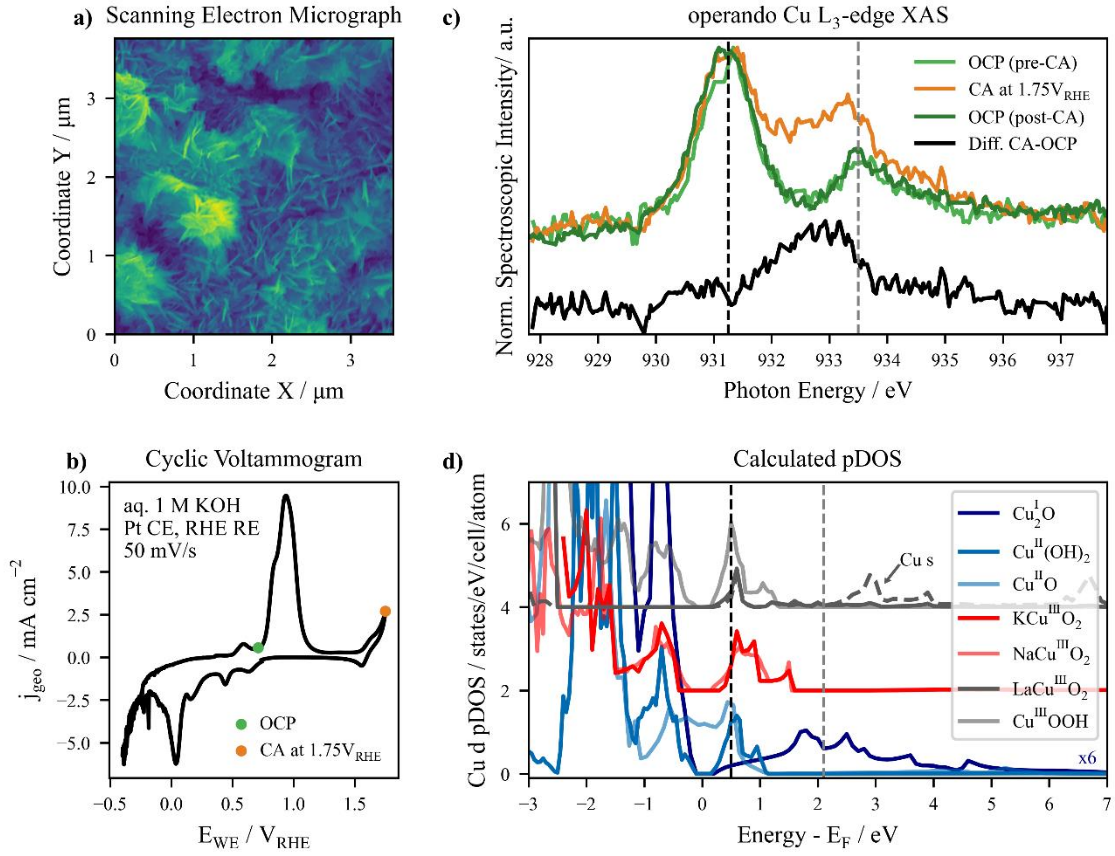

Addition of new section in SI (pp.7-8) in red:

Considerations about XAS at OCP

In **figure 1c**, XAS during Chrono-Amperometry (CA) at 1.75 V_{RHE} and at Open Circuit Potential (OCP) conditions (before and after CA) are shown. Understanding the behaviour of the catalyst during the post-CA OCP is important to correlate the probed chemical species with the electrochemical response presented. Therefore, the relaxation of the OCP after the exposition of the catalyst to high anodic potentials during the potential hold is investigated here on a Cu electrode (250 um Cu foil), mirroring the electrochemical protocol used in our operando XAS study.

Figure S7 bottom shows the temporal evolution of OCP after a 300 s potential hold at 1.79 V_{RHE} (current density during CA shown in the **top panel**). It can be observed how OCP consistently relaxes back to its original value of approximately 0.75 V_{RHE} after the applied potential is removed. OCP decays close to the initial OCP value within ~60 seconds and fully returns to the baseline (~0.75 V_{RHE}) within 120 seconds. Since our operando XAS measurements at OCP were taken after a 60 seconds relaxation period (and each spectrum acquisition also takes approximately 60 seconds), the spectra recorded at OCP reliably represent the material at conditions similar to its initial OCP state.

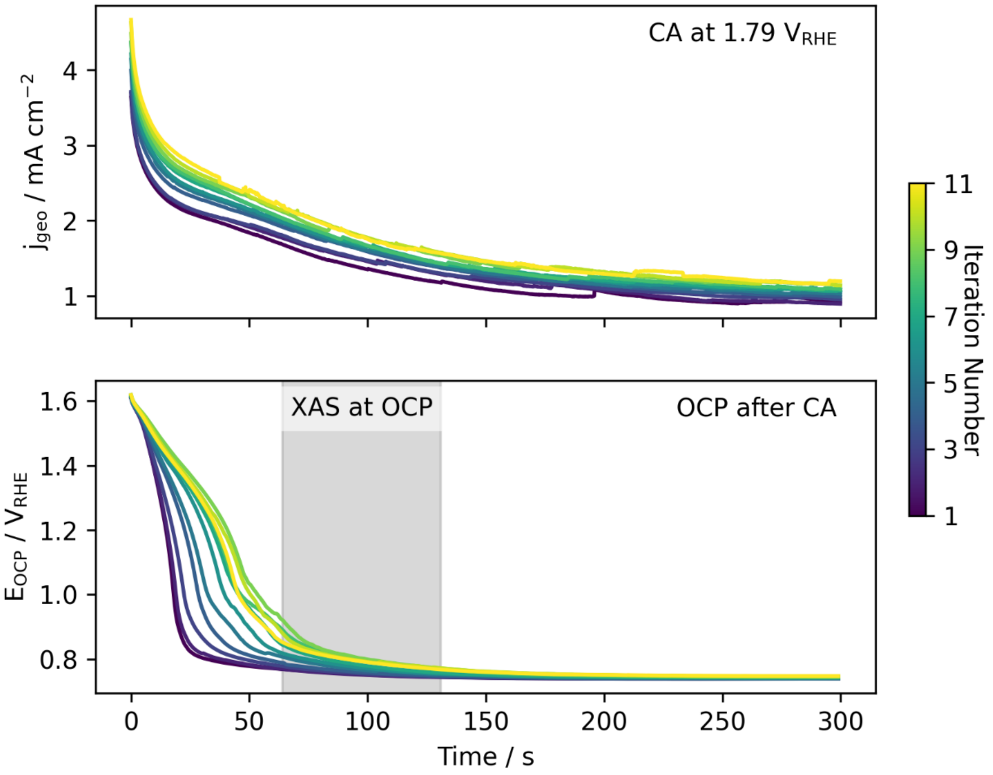

Figure S7: *Top panel:* Chronoamperometric (CA) current response during a 300 s potential hold at 1.79 V_{RHE}, showing the current density evolution under OER conditions. *Bottom panel:* Temporal evolution of the open circuit potential (OCP) following the CA step. After the applied potential is removed, the OCP decays toward its initial value of ~0.75 V_{RHE} within ~60 seconds and fully recovers its original value after ~120 seconds. For completeness, the period in which the XAS at OCP is measured is highlighted in gray.

<u>Reviewer: 2</u>

Comments:

This research article has focused on High-valent intermediate observed in a Cu-based OER electrocatalyst by XAS. The authors have reported material synthesis, characterization, and in situ XAS. However, based on the current version and limited novelty, a major revision is needed before publishing in the Journal Physical Chemistry Letters. See comments below.

Comments and questions

Q1: More characterization on the synthesized Cu oxides should be done, like XRD, FT-IR, XPS ect.

We thank the reviewer for the suggestion to include additional characterization. Although our primary method, *operando* XAS, is particularly well-suited for probing the oxidation state and local coordination of copper species in its native electrochemical medium —in our case, in 1 M KOH-, we have performed additional ex-situ characterizations of the high surface area electrocatalyst. To complement the XAS measurements at open circuit potential (OCP), we measured ex-situ X-ray photoelectron spectroscopy (XPS) and Scanning Electron Microscopy (SEM) with Energy Dispersive X-ray Spectroscopy (EDX) on the high surface area electrocatalyst, as described in the new section in SI “Ex-situ characterization of the Cu-based electrocatalyst”

Changes in the manuscript (p.6) in red:

(…) arising from the anodization synthesis of the electrocatalyst, as previously reported [25,36] and further confirmed by X-ray Photoelectron Spectroscopy (See Ex-situ characterization of the Cu-based electrocatalyst in Supplementary Information). Upon applied potential (1.75 V_{RHE}), (…)

Addition of new section in SI (pp.5-7) in red:

***Ex-situ* characterization of the Cu-based electrocatalyst**

For the *ex-situ* characterization, a high surface Cu-based electrocatalyst was prepared on a Pt-coated Si wafer by electrodepositing copper on the Pt current collector by 100 s Chronoamperometry (CA) at -0.1 V_{RHE} in aq. 0.1M $CuSO_4$ followed by the oxidation of the film by an 80 min CA at 1.8 V_{RHE} in aq. 1M KOH, as displayed in **Figure S3a**. A Cyclic Voltammogram (CV) in 1M KOH with 50 mV/s scan rate is used to characterize the produced electrocatalyst, as shown in **Figure S3b.**

The ex-situ characterization of the electrocatalyst was performed after removing the electrode from the electrochemical medium and rinsing with DI water.

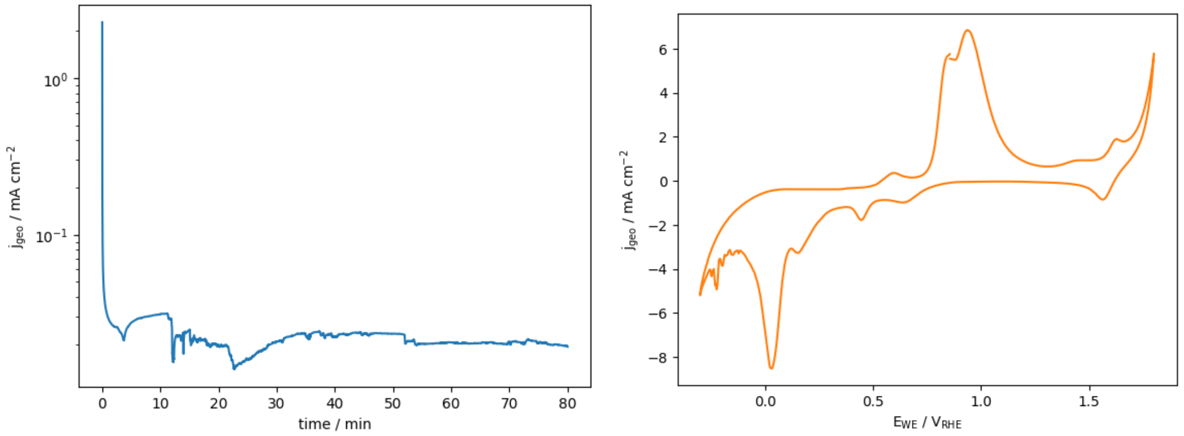

Figure S3: **a)** Current profile of the electrodeposited Cu thin film in aq. 1 M KOH using CA at 1.8 V_{RHE} during 80 min for the preparation of the high surface area Cu catalysts. **b)** CV of the high surface Cu-based electrocatalyst used for the ex-situ characterization. CV were recorded in aq. 1M KOH with the scan rate of 50 mV/s. Pt mesh was used as CE, while a RHE was used as reference electrode.

X-ray Photolectron Spectroscopy

Ex-situ surface analysis of the material was carried out by Al K_a XPS. In **Figure S4a**, a survey of the electrode is shown, where the characteristic peaks of copper and oxygen can be observed, associated with the electrocatalyst. The peaks of potassium (K) arise from remaining traces of the electrolyte (aq. 1M KOH) on the electrode are also detected (see SEM-EDX for further details), as well as the signal from the Pt current collector.

In the detailed core-level spectrum of Cu 2p depicted in **Figure S4b**, the characteristic strong

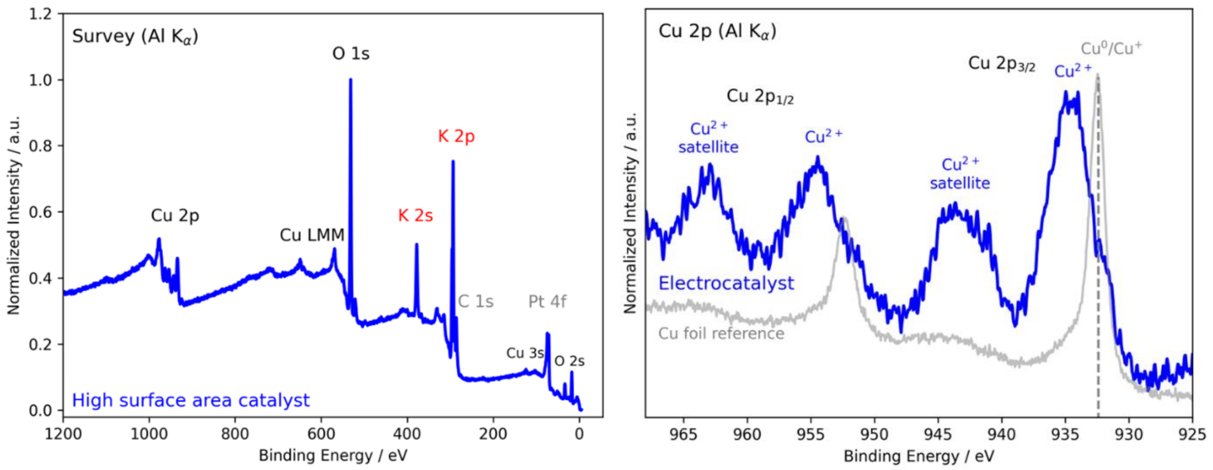

satellite peaks of Cu^{2+} are clearly visible, as well as the chemical shift associated with this oxidation state (Cu foil as reference). These observations further support the findings by XAS that the species present at the surface of the electrocatalyst are of Cu(II) nature, likely $CuO/Cu(OH)_2$.

Figure S4: **a)** Survey and **b)** Cu 2p core-level spectra of the high surface Cu-based electrocatalyst. For comparison purposes, the spectrum of a Cu foil has been included in the Cu 2p spectrum.

SEM and EDX-SEM

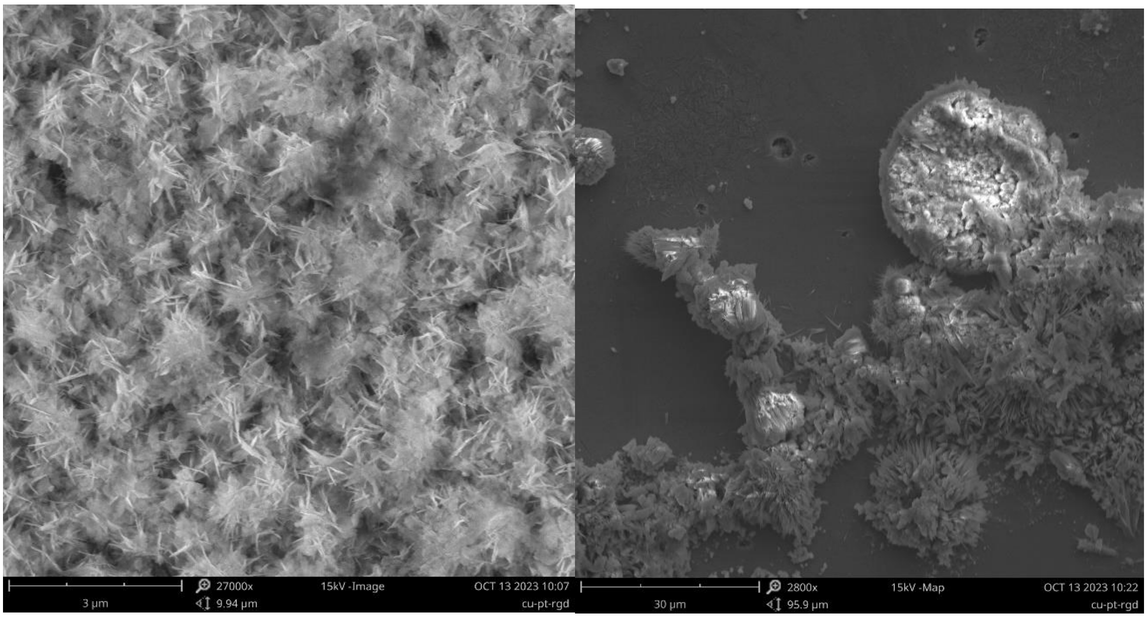

Figure S5: Low magnification 15 kV SEM images of the high surface area Cu-based electrocatalyst

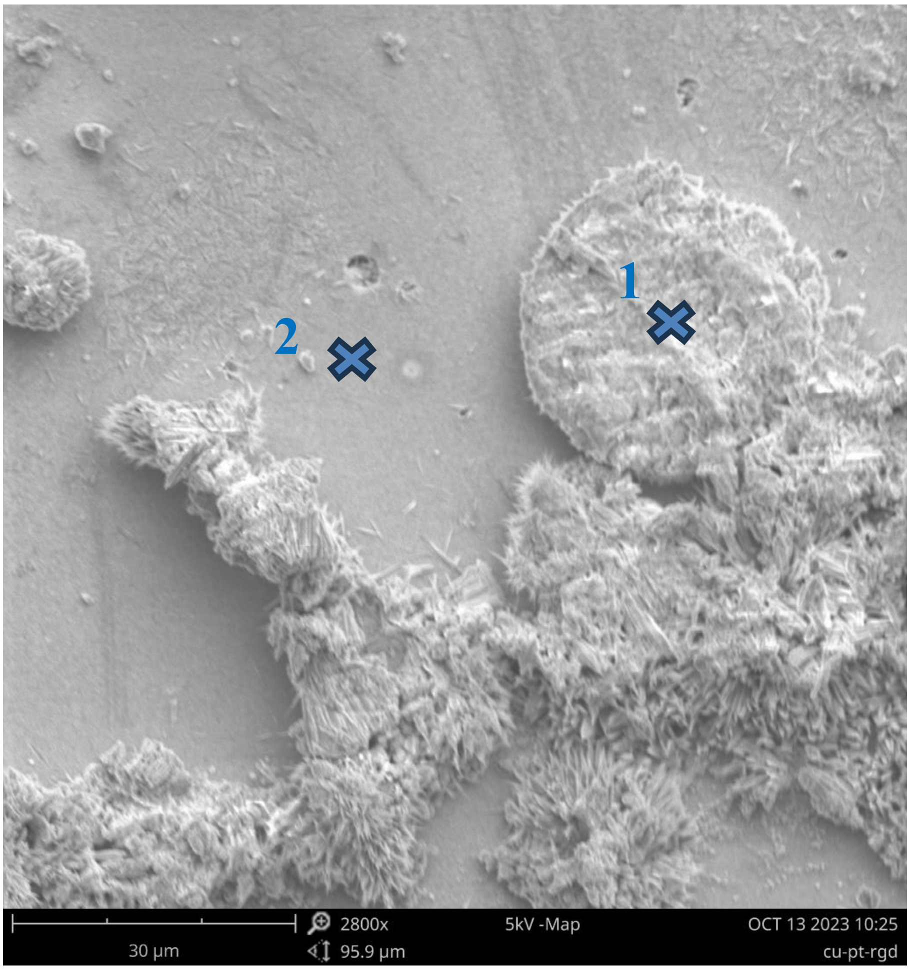

Figure S6: 5 kV SEM image used for the EDX analysis of the high surface area Cu-based electrocatalyst. The 2 analyzed sample spots are shown as crosses.

Element	Spot 1 (5 kV)	Spot 1 (15 kV)	Spot 2 (5 kV)	Spot 2 (15 kV)
Cu	56.14	71.41	4.33	7.05
O	43.57	17.61	8.24	6.33
K	-	10.27	79.15	1.62
Pt	0.30	-	6.60	36.03
Si	-	0.72	1.68	48.96

Table S2: SEM-EDX at 5 and 15 kV: Elemental analysis of 2 sample spots of the electrode, corresponding to: 1) areas with high concentration of the high surface area Cu-based electrocatalyst and 2) less covered areas. The atomic concentrations are shown for all the elements of the electrode.

Q2: The post-catalytic SEM data should be added to demonstrate the structural stability.

We thank the reviewer for the suggestion to include additional post-catalysis characterization. However, due to the specific design and constraints of the operando soft XAS experiments, it is not feasible to recover the electrode material for ex-situ analysis after the experiment. The electrodes used are very thin to ensure compatibility with the short attenuation length of soft X-rays and under the electrochemical conditions applied in this study—particularly at potentials exceeding the Cu(II)/Cu(III) redox transition—the copper-based catalyst undergoes harsh dissolution, resulting in an complete dissolution of the high surface area Cu-based electrocatalyst at the end of each experiment, as shown in Figure S8 in SI. In summary, following the operando measurements, no detectable amount of material remains on the SiN window to allow for meaningful post-mortem SEM or other surface characterization.

Q3: The Electrochemically active surface area (ECSA) of Cu oxide electrocatalyst is missing. Post-catalytic ECSA should also be reported.

Similarly, post-catalysis ex-situ ESCA measurements cannot be performed on the operando electrodes due to the same limitations described above.

However, to gain insight into changes in electrochemical surface area induced by high-potential treatments, we performed complementary experiments on bulk Cu foil as a model system. Specifically, we applied chronoamperometric (CA) holds at high potentials (1.79 V_{RHE}) on the Cu foil and recorded cyclic voltammograms afterwards. We observed that voltametric charge increases progressively with prolonged high-potential treatment, which we interpret as an increase in the electrochemical surface area.

As a semi-quantitative analysis, we plotted the current density of the redox peak associated with the Cu(I)/Cu(II) transition as a function of the number of CA cycles applied (each cycle consisting of a

300-second CA hold). These data, included in the revised Supporting Information, provide useful indirect evidence of surface area changes under conditions relevant to our operando study.

Changes and additions in SI (pp.2-3) in red:

In addition, the increase of the surface area of the Cu-based electrocatalyst at the aforementioned conditions has been electrochemically studied on a Cu electrode (250 um Cu foil) by applying consecutive 300 s potential holds at OER-relevant potentials (CA at 1.79 V_{RHE}) and measuring characterization Cyclic Voltammograms (CV) after each iteration, as shown in **figure S2**. The evolution of the electrocatalyst area with the cycle number can be evaluated by the current density at the maximum of the Cu(I)/Cu(II) anodic redox peak at 0.87 V_{RHE}, as depicted in the **bottom panel**. The electrochemically active surface area keeps growing as long as the potential hold is maintained at 1.79 V_{RHE} (number of cycles), likely related with an increase of the surface-to-bulk ratio, as complementarily observed by in-situ XAS (**figure S1**).

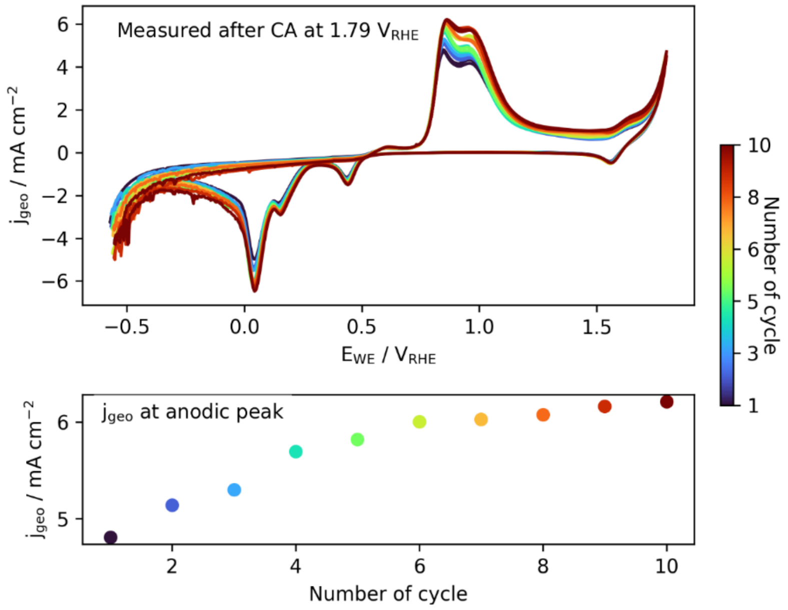

Figure S2. *Top panel:* Cyclic voltammograms (CVs) recorded in aq. 1M KOH at 50 mV/s on a 250 μm Cu foil after consecutive 300 s potential holds at 1.79 V_{RHE}. CE: Pt mesh, RE: RHE. *Bottom panel:* Evolution of the anodic peak current density at ~0.87 V_{RHE} (Cu(I)/Cu(II) transition) as a function of the number of CA cycles. This redox feature serves as an electrochemical proxy for the active surface area, which increases progressively with each potential hold and suggests a continuous increase in the surface-to-bulk ratio.

Q4: Lower magnification SEM images of Cu oxide electrocatalyst should be provided to see the homogeneity of the electrode surface.

Lower magnification SEM and SEM-EDX micrographs have been added to SI in the new section "Ex-situ characterization of the Cu-based electrocatalyst"

Addition of new section in SI (pp.5-7) in red:

***Ex-situ* characterization of the Cu-based electrocatalyst**

(..)

SEM and EDX-SEM

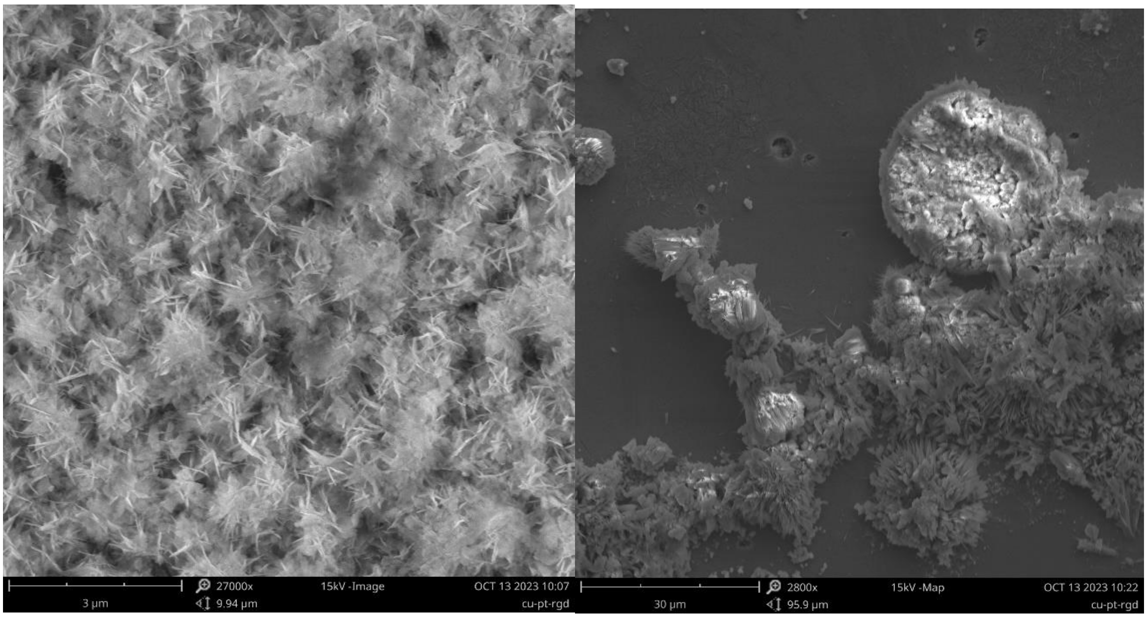

Figure S5: Low magnification 15 kV SEM images of the high surface area Cu-based electrocatalyst

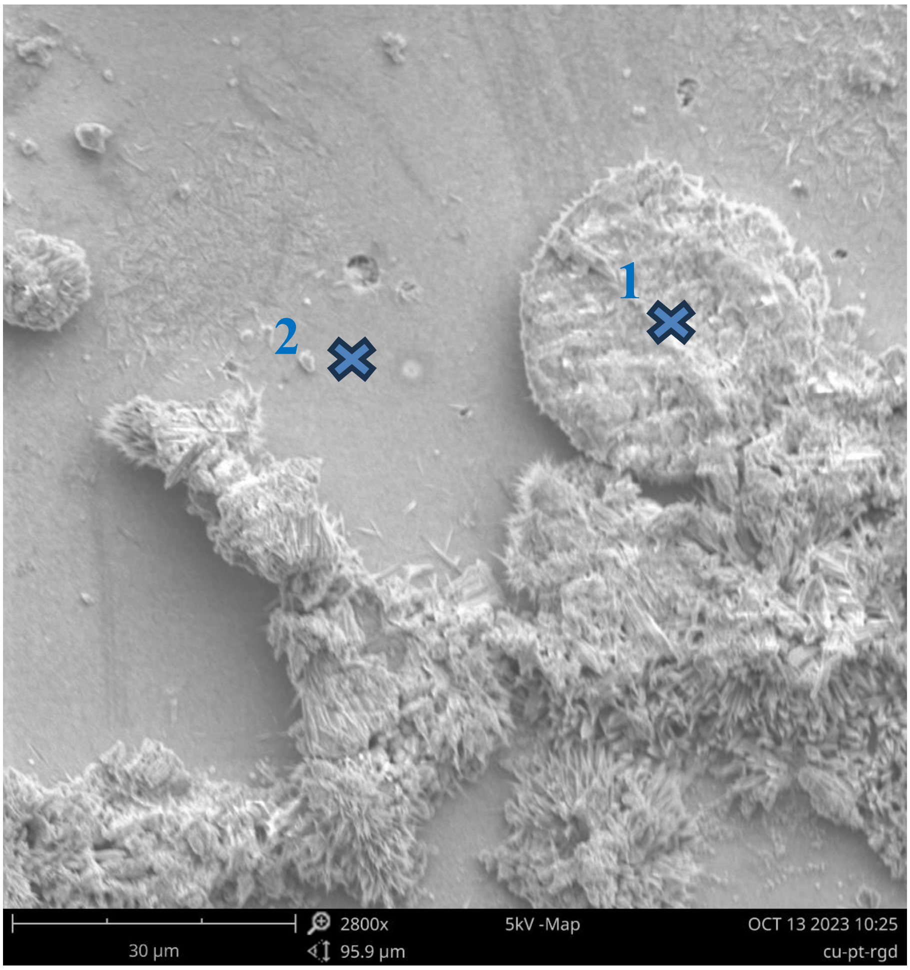

Figure S6: 5 kV SEM image used for the EDX analysis of the high surface area Cu-based electrocatalyst. The 2 analyzed sample spots are shown as crosses.

Element	Spot 1 (5 kV)	Spot 1 (15 kV)	Spot 2 (5 kV)	Spot 2 (15 kV)
Cu	56.14	71.41	4.33	7.05
O	43.57	17.61	8.24	6.33
K	-	10.27	79.15	1.62
Pt	0.30	-	6.60	36.03
Si	-	0.72	1.68	48.96

Table S2: SEM-EDX at 5 and 15 kV: Elemental analysis of 2 sample spots of the electrode, corresponding to: 1) areas with high concentration of the high surface area Cu-based electrocatalyst and 2) less covered areas. The atomic concentrations are shown for all the elements of the electrode.

Q5: One relevant work should be cited in this work:

ACS Appl. Mater. Interfaces, 2024, 16, 35793–35804, DOI: 10.1021/acsami.4c06365

It has been added in page 3 as REF 21, after "(…) To resolve these ambiguities, operando X-ray absorption spectroscopy (XAS) emerges as an ideal tool (…)"
